# Longitudinal patterns of fentanyl utilisation (medical versus illicit sources) in the United States 2015–2023: a retrospective cohort study

**DOI:** 10.1016/j.eclinm.2026.104141

**Published:** 2026-08-14

**Authors:** Seungyeon Lee, Wenyu Song, David W. Bates, Richard D. Urman, Ping Zhang

**Affiliations:** a Department of Computer Science and Engineering, The Ohio State University, Columbus, OH, USA; b Department of Biomedical Informatics, The Ohio State University, Columbus, OH, USA; c Department of Medicine, Brigham and Women’s Hospital, Harvard Medical School, Boston, MA, USA; d Department of Anesthesiology, College of Medicine, The Ohio State University, Columbus, OH, USA

**Keywords:** Fentanyl exposure, Illicit fentanyl use, Opioid overdose, Urine drug testing, Electronic health records

## Abstract

**Background:**

Over the last decade, fentanyl use in the U.S. has experienced a dramatic shift, largely driven by a rise in illicit fentanyl and its role in the opioid overdose crisis. Characterizing individual-level illicit fentanyl exposure poses significant challenges, as such use often occurs outside clinical settings and is not directly captured in clinical data, making it difficult to measure its true scale and impact.

**Methods:**

We conducted a retrospective cohort study using Epic Cosmos, a large U.S. electronic health record dataset comprising over 300 million patients across inpatient and outpatient settings nationwide (January 1, 2015, to December 31, 2023). The dataset provides individual-level clinical data, including diagnoses, medication records, and laboratory testing data, enabling longitudinal characterization of fentanyl exposure. To infer sources of fentanyl exposure, we linked urine drug testing (UDT) results to fentanyl medication records using a sequential time window screening method (e.g., UDT positive with pre-fentanyl records or without). Temporal and Cox proportional hazards analyses were used to examine longitudinal patterns and risks of opioid-related harmful outcomes associated with medical-source versus illicit-source fentanyl exposure. Regional variation was explored. Confounding was adjusted using stabilized inverse probability weighting based on demographics, social vulnerability index, and 37 baseline conditions. Subgroup analyses tested effects of underlying clinical burden. Sensitivity analyses evaluated alternative UDT screening windows and follow-up periods to assess sensitivity to exposure and outcome definitions.

**Findings:**

Our study cohort included 295,728 patients, consisting of 85,535 (28.9%) in the medical-source cohort (MSC) and 210,193 (71.1%) in the illicit-source cohort (ISC). From 2015 to 2023, the prevalence of nonfatal opioid overdose was consistently higher in the ISC. Overdose prevalence in the ISC increased markedly over time, reaching 18.6%, whereas only a modest increase was observed in the MSC, reaching 4.3%. For opioid dependence and abuse, the ISC had higher prevalence, and steeper year-over-year increases, versus the MSC until 2020. After 2020, prevalence in the ISC declined, particularly for dependence, but remained consistently higher than in the MSC throughout the study period. Region-stratified temporal patterns followed the overall cohort-level patterns. Illicit-source fentanyl initiation was associated with significantly elevated risk of 30-day opioid-related harmful outcomes, with adjusted hazard ratios of 2.99 (95% CI, 2.71–3.29; *P* < 0.001) for overdose, 1.96 (95% CI, 1.84–2.10; *P* < 0.001) for abuse, and 3.05 (95% CI, 2.89–3.22; *P* < 0.001) for dependence, compared with medical-source initiation, after confounding adjustment. Across subgroups stratified by clinical conditions, illicit-source initiation was consistently associated with increased risk of outcomes.

**Interpretation:**

Illicit-source fentanyl exposure is associated with a markedly higher risk of opioid-related harmful outcomes than medical-source exposure, providing evidence that illicit fentanyl is a driver of adverse patient outcomes. Whilst acknowledging the limitations of this analysis, these findings underscore the substantial contribution of illicit fentanyl to the ongoing opioid epidemic and highlight the need for deeper investigation of how medical and illicit fentanyl use interact over time. Further research is warranted.

**Funding:**

National Institute on Drug Abuse (NIDA) and National Science Foundation (NSF).

**Research in context**

**Evidence before this study**

Illicit fentanyl has emerged as a dominant driver of opioid-related overdose deaths in the U.S., yet its true scale and impact remain difficult to quantify because illicit use occurs largely outside formal healthcare systems. Urine drug testing (UDT) serves as a valuable diagnostic tool to detect nonprescribed or illicit drug exposure. We searched PubMed on September 2025 using the terms (“fentanyl”) and (“urine drug testing” or “urine drug test”) within the past 10 years. The search retrieved 63 studies, but only 7 were relevant to our research. These studies have leveraged UDT data to track trends in illicit fentanyl exposure, including changes in nonprescribed fentanyl co-detection with other substances, and to estimate overdose burden. However, few of these studies have systematically distinguished fentanyl exposure occurring within healthcare settings from exposure likely occurring outside medical supervision. Consequently, the relative contributions and impacts of medical-versus illicit-source fentanyl on opioid-related harmful outcomes over time remain incompletely understood.

**Added value of this study**

Using a large, national-scale electronic health record dataset from Epic Cosmos covering all 50 U.S. states from 2015 to 2023, we examined longitudinal patterns and short-term risks of opioid-related harmful outcomes associated with sources of fentanyl exposure. By linking UDT data with documented fentanyl medication records using a sequential time-window design, we inferred medical-source and potential illicit-source fentanyl exposure. To our knowledge, this is one of the first and largest observational studies to quantify the scale and impact of illicit-source fentanyl exposure on the national level and among major geographic regions in the U.S. These findings demonstrate the growing contribution of illicit fentanyl to the opioid epidemic and highlight the need to better understand interactions between medical and illicit fentanyl use over time.

**Implications of all the available evidence**

Despite evidence on the harms associated with illicit fentanyl use, there is no systematic national-level analysis examining how the source of fentanyl exposure influences opioid-related harmful outcomes. Though there are limitations to consider, our findings suggest that illicit-source fentanyl exposure is associated with substantially greater and persistently increasing opioid-related harms compared with medical-source exposure. These results suggest that strategies focused primarily on regulating medical opioid use are insufficient to address the current overdose crisis. Integrating clinical testing data with medication records, as demonstrated in this study, provides a scalable, data-driven framework to monitor illicit fentanyl exposure and inform timely public health responses to the evolving epidemic. Further research is required.

## Introduction

Opioids represent among the most powerful analgesics available and are widely used for acute and chronic pain.^1^ Although highly effective for pain relief, opioids have serious side effects and substantial risks, including misuse, dependence, and overdose.^2^ Overdose deaths involving prescription opioids increased substantially beginning in the 1990s but have declined in recent years. Since 2013, overdose deaths involving synthetic opioids, particularly illicitly manufactured fentanyl and fentanyl analogues, have risen sharply.^3^ In 2023, approximately 105,000 people in the U.S. died from drug overdose,^3^ and nearly 73,000 of these deaths involved synthetic opioids, primarily fentanyl,^4^ accounting for approximately 92% of all opioid-involved overdose deaths. These trends reflect the evolving nature of the opioid crisis, with recent increases driven by the growing availability of high-potency synthetic opioids.

Fentanyl plays a critical role as a potent synthetic opioid used for managing severe pain, particularly in patients with cancer and in post-surgical settings,^4^^,^^5^ but its high potency also increases the risk of overdose, making its use tightly regulated. Fentanyl is 100 times more powerful than morphine and heroin,^6^ and its analogues can be even more potent. Even small doses can lead to fatal overdose, and both non-fatal and fatal overdoses involving fentanyl have risen substantially over the past decade.^7^ Legal pharmaceutical fentanyl is prescribed or administered under medical supervision to treat severe pain.^8^ In contrast, illicitly manufactured fentanyl is distributed through illicit drug markets,^9^^,^^10^ and has emerged as a major driver of recent fentanyl-related overdoses^11^ and overdose deaths.^12^ Individuals with opioid use disorder frequently use illicit fentanyl, markedly increasing their risk of overdose and death.^13^

Despite extensive evidence on the adverse outcomes associated with illicit fentanyl use, it is difficult to track individual-level illicit fentanyl exposure because such use often occurs outside healthcare systems^14^ and is not directly captured in clinical data, limiting accurate estimation of its true scale and impact. Urine drug testing (UDT) serves as a valuable diagnostic tool across medical disciplines, including addiction medicine,^15^ and plays a critical role in detecting nonprescribed or illicit drug use.^16^^,^^17^ Previous studies have used UDT data to track drug trends, including changes in nonprescribed fentanyl co-detection with cocaine or methamphetamine,^18^^,^^19^ and to estimate overdose deaths using aggregated UDT data.^17^ However, very few of these studies have efficiently differentiated between medical-source and illicit-source fentanyl exposure, leaving a critical gap in understanding how the source of fentanyl exposure influences opioid-related harmful outcomes.

Leveraging patient-level electronic health records (EHRs), we inferred potential illicit-source fentanyl (non-medical) exposure by linking UDT results with documented fentanyl-related medication records using a sequential time-window screening method to differentiate medical- and illicit-source fentanyl exposure. We characterized longitudinal and regional patterns in opioid-related harmful outcomes stratified by the source of fentanyl exposure and estimated the risk of the outcomes associated with illicit-source fentanyl exposure.

## Methods

### Study design and ethics

We performed a retrospective cohort study using large-scale, longitudinal, individual-level healthcare data from the Epic Cosmos,^20^ a U.S. national clinical dataset created and managed by the Epic Systems Corporation that serves as the EHR vendor. The Cosmos integrates both inpatient and outpatient charts into a single, comprehensive record, including as patients move between health systems. It includes over 300 million patients from 1762 hospitals and 40,700 clinics from all 50 U.S. states, and is broadly representative of the U.S. Census for age, race, ethnicity, coverage, and the Social Vulnerability Index (SVI).^20^

**Fig. 1:**
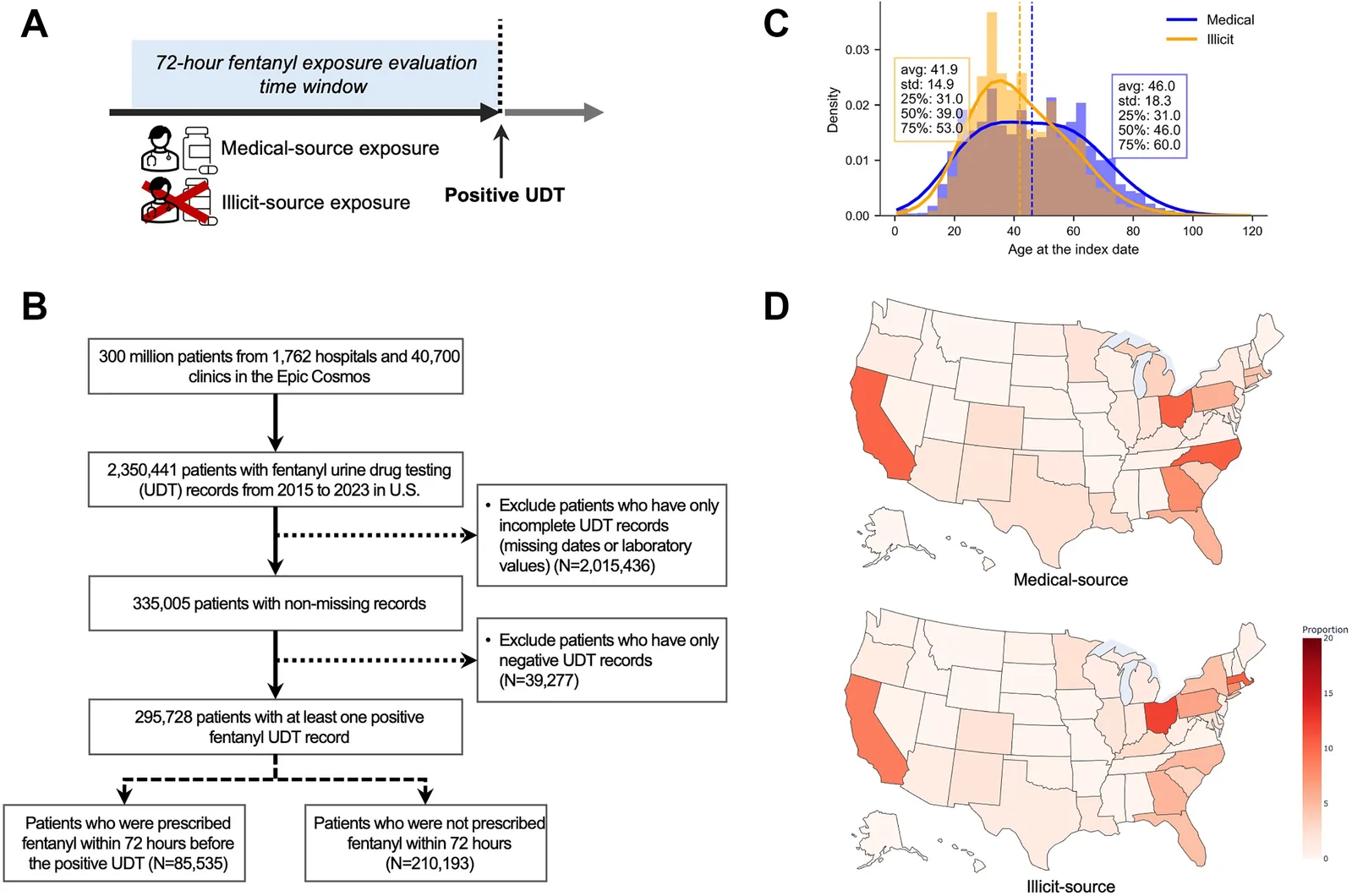
**Study design and demographic characteristics of medical- and illicit-source fentanyl exposure cohorts.** A. identification strategy of the source of fentanyl exposure; B. a flowchart of patient inclusion and exclusion; C. age distributions at the index date; and D. geographic distributions for the medical- and illicit-source fentanyl exposure cohorts. The index date was defined as the initial fentanyl exposure, either the date of the first fentanyl medication record or the first positive UDT result, whichever occurred first. UDT = Urine drug testing, Medical = medical-source fentanyl exposure cohort, Illicit = illicit-source fentanyl exposure cohort.

We identified an initial cohort of 2,350,441 patients with at least one fentanyl UDT record from Jan 1, 2015 to Dec 31, 2023. Of these, about 295,728 patients met the study inclusion criteria (Fig. 1b). The data utilized for this study included comprehensive patient-level information, including demographics, medication records, diagnosis records, and laboratory results from inpatient, outpatient, and emergency department encounters.

This study used secondary, de-identified data and was therefore exempt from ethics committee approval. The requirement for written informed consent was waived owing to the retrospective study design and the use of de-identified secondary data.

### Key variables

Urine drug testing (UDT) data, a key diagnostic tool in addiction medicine,^15^ was used to identify and track fentanyl exposure. All fentanyl UDT records were extracted, including fentanyl or norfentanyl test results by screen or confirmatory methods. Records were removed if the specimen collection date (or the best available proxy date) was missing or if both the quantitative results with units and qualitative results (i.e., positive or negative, or abnormal) were unavailable. Only records that met all completeness requirements were retained for analysis. All quantitative measurements were standardized into a consistent unit (ng/mL) and classified using a predefined threshold of 3 ng/mL,^21^ with values at or above this threshold classified as positive for fentanyl exposure and lower values classified as negative.

To infer medical-source and potential illicit-source (non-medical) fentanyl exposure, UDT results were linked to documented fentanyl medication records using a sequential time-window screening method (Fig. 1a). We identified fentanyl medication records whose service date and days’ supply (when available) overlapped a 72-h window preceding the positive UDT date, consistent with the typical 2–4 day urine detection period for opioids.^22^ Positive UDTs with a linked medication record were attributed to medical-source exposure, whereas positive UDTs without a linked record were categorized as potential illicit-source exposure, indicating fentanyl exposure outside healthcare settings. The index date was defined as the initial fentanyl exposure, either the date of the first fentanyl medication record or the first positive UDT result, whichever occurred first. Fentanyl medication records included medication orders and medication administration records (Supplementary Table S3).

### Participants

The study cohort included all individuals with at least one positive fentanyl UDT result between 2015 and 2023. They were classified into two subcohorts based on the inferred source of fentanyl exposure; individuals for whom all positive UDT results were temporally linked to fentanyl medication records were categorized as the medical-source fentanyl exposure cohort, whereas individuals for whom any positive UDT result lacked a temporally linked record were classified as the illicit-source fentanyl exposure cohort. The illicit-source cohort may therefore include individuals with exclusively illicit fentanyl exposure as well as those with both medical- and illicit-source exposures over time, including transitions between exposure sources.

### Cohort characteristics

Baseline cohort characteristics were examined to assess their association with the source of fentanyl exposure. Demographic variables included age on the index date, sex, marital status, race/ethnicity, and state of residence. States were grouped into four major U.S. Census regions—Northeast, Midwest, South, and West—for all stratified analyses (Supplementary Table S1). Social context was also evaluated using the Social Vulnerability Index (SVI), which is based on percentile rankings by the ZIP code and ranges from 0 to 1, with higher values indicating greater vulnerability, and serves as a standardized, multidimensional measure of area-level social context. Fentanyl-related characteristics included the number of fentanyl medication records, the number of positive UDTs, and the mean of quantitative UDT values (Avg UDT). To ensure data reliability, the top 1% of UDT values were excluded from summary statistics to minimize the influence of potential outliers.

Baseline clinical conditions were assessed during the three months before the index date (the initial fentanyl exposure). A total of 40 clinical conditions were examined, including 24 chronic diseases (e.g., cancer, diabetes, and hypertension), five mental health disorders (e.g., anxiety, bipolar, and depression), three opioid-related harmful outcomes (overdose, abuse, and dependence), and eight substance use disorders (e.g., stimulant, alcohol, cannabis, and tobacco), all defined using International Classification of Diseases (ICD) codes (Supplementary Table S2). Opioid overdose was defined as nonfatal events using all ICD-10 subcodes under T40.0–T40.4 and T40.6, excluding adverse effect and underdosing codes. Fatal overdose events were not captured, as cause-of-death information (ICD: X40–44, X60–64, X85, and Y10–14) is not available in the dataset. Clinical conditions were identified using Epic Cosmos terminology-based mappings to ensure consistent condition definitions throughout the study period.

### Statistical analysis

We compared baseline characteristics between the medical- and illicit-source cohorts and across region-stratified subgroups within each cohort. Variables included demographics, SVI measures, fentanyl-related factors, and the prevalence of the baseline clinical conditions. Most continuous and count variables were non-normally distributed; therefore, non-parametric tests were prespecified. We used two-sided Mann–Whitney U tests for pairwise comparisons and Kruskal–Wallis tests for regional comparisons. All tests were considered statistically significant at *P* < 0.05.

To evaluate the causal association between illicit-source fentanyl initiation and 30-day opioid-related harmful outcomes, including nonfatal opioid overdose, opioid abuse, and opioid dependence, we estimated hazard ratios (HRs) using Cox proportional hazards models.^23^ The exposure was illicit-source fentanyl initiation at the index date (the initial fentanyl exposure date). Individuals with a positive UDT at the index date were classified as having illicit-source fentanyl initiation (exposure), whereas those with a fentanyl medication record at the index date were classified as having medical-source fentanyl initiation (no exposure). The outcome was the occurrence of an opioid-related harmful outcome (e.g., opioid dependence) within 30 days after the index date. Individuals who experienced the outcome were coded as events at the observed time-to-event, whereas those without the outcome were coded as censored at day 30. Individuals with any prior opioid-related harmful outcomes were excluded for the association analysis to ensure assessment of incident outcomes.

Confounding was adjusted using stabilized inverse probability weighting (IPW) based on demographics, SVIs, and 37 baseline conditions (excluding opioid-related outcomes) measured before the index date. Other opioid prescriptions were not included as covariates, as the primary exposure of interest was the source of fentanyl exposure. Propensity scores were estimated by logistic regression of exposure on these covariates, with 3-fold cross-validation for training and evaluation. Post-weighting covariate balance was assessed using absolute standardized mean differences (SMD), a commonly used metric for evaluating between-group differences, with |SMD| ≤0.10 indicating adequate balance. Analyses were considered balanced if the proportion of unbalanced covariates was ≤ 2% of all covariates.^24^

An IPW-weighted Cox model was fitted in the entire study cohort to estimate adjusted HRs for illicit-source fentanyl initiation, after confirming covariate balance. We additionally conducted analyses restricted to the illicit-source cohort alone to further evaluate associations within a higher-risk subpopulation; all patients in the medical-source cohort were classified as having non-illicit-source fentanyl initiation.

Post-hoc subgroup analyses were conducted to examine whether associations varied by underlying clinical conditions, including substance use disorders, mental health conditions, and chronic conditions (ICD-based definitions in Supplementary Table S2). For each clinical category, patients were grouped by the presence or absence of any prior condition, and analyses were performed within each subgroup using the same analytic framework as in the primary analysis. Post-hoc sensitivity analyses evaluated alternative UDT screening windows (48, 72, and 96 h) and follow-up periods (30, 60, 90, and 180 days) to assess sensitivity to exposure and outcome definitions (Supplementary Tables S9 and S10). All statistical analyses were conducted using Python (version 3.12), SciPy, and Causallib^25^ libraries.

### Temporal and regional patterns

We examined longitudinal patterns in disease prevalence within the medical- and illicit-source fentanyl cohorts from 2015 to 2023 to enable year-over-year comparisons of disease burden and its evolution over time. Region-stratified analyses were additionally performed to characterize geographic variation in these patterns. For each year, we calculated the percentage of patients diagnosed with each outcome of interest. To ensure temporal validity, we included only individuals whose index date occurred within the corresponding year, thereby assessing disease prevalence among individuals with confirmed fentanyl initiation during that year.

### Role of the funding source

The funder had no role in the study design, data collection, data analysis, data interpretation, or writing of the manuscript.

## Results

### Descriptive summary of the study cohort

From an initial cohort of 300 million patients, we developed a study cohort of 295,728 patients from January 1, 2015, to December 31, 2023 (Table 1), including patients from the South (33.3%), Northeast (28.6%), Midwest (21.4%), and West (16.6%). The cohort selection pipeline is summarized in Fig. 1b. Of these, 85,535 (28.9%) patients were the medical-source fentanyl exposure cohort, while 210,193 (71.1%) patients were included in the illicit-source cohort.

**Table 1:** Summary statistics on the entire cohort, and the medical- and illicit-source fentanyl exposure cohorts.

Variables	Total	Medical-source	Illicit-source	*P* value^a^
Demographics				
Patients, No. (%)	295,728	85,535 (28.9%)	210,193 (71.1%)	NA
Male, No. (%)	176,107 (59.6%)	51,163 (59.8%)	124,944 (59.4%)	0.061
Female, No. (%)	119,621 (40.4%)	34,372 (40.2%)	85,249 (40.6%)	0.061
Married, No. (%)	52,419 (17.7%)	22,458 (26.3%)	29,961 (14.3%)	<0.001
Age, mean (SD)	43.229 (16.32)	46.079 (18.39)	41.99 (15.168)	<0.001
Age, median (IQR)	41.0 (31.0, 55.0)	46.0 (31.0, 60.0)	39.0 (31.0, 53.0)	NA
Race, No. (%)				
White	181,228 (61.3%)	51,283 (60.0%)	129,945 (61.8%)	NA
Black or African American	65,776 (22.2%)	20,393 (23.8%)	45,383 (21.6%)	NA
American Indian or Alaska native	8609 (2.9%)	2026 (2.4%)	6583 (3.1%)	NA
Asian	3358 (1.1%)	1377 (1.6%)	1981 (0.9%)	NA
Native Hawaiian or other Pacific Islander	974 (0.3%)	268 (0.3%)	706 (0.3%)	NA
Other race	14,518 (4.9%)	4073 (4.8%)	10,445 (5.0%)	NA
SVI, mean (SD)				
Overall	0.681 (0.271)	0.677 (0.272)	0.683 (0.27)	<0.001
Socioeconomic	0.635 (0.288)	0.635 (0.288)	0.635 (0.288)	0.523
Household characteristics	0.612 (0.288)	0.622 (0.282)	0.608 (0.291)	<0.001
Housing type transportation	0.69 (0.251)	0.672 (0.256)	0.697 (0.248)	<0.001
Racial ethnic minority	0.692 (0.241)	0.685 (0.246)	0.695 (0.239)	<0.001
Fentanyl				
Medication record count, mean (SD)	14.85 (43.757)	32.723 (63.352)	7.577 (29.625)	<0.001
Urine drug testing (UDT) count, mean (SD)	2.465 (6.045)	1.405 (1.858)	2.897 (7.026)	<0.001
Avg UDT value, mean (SD)	77.661 (101.559)	47.542 (61.187)	87.428 (112.875)	<0.001
Clinical conditions^b^				
Cancer, No. (%)	6059 (2.0%)	2732 (3.2%)	3327 (1.6%)	<0.001
Cardiovascular disease	44,709 (15.1%)	14,660 (17.1%)	30,049 (14.3%)	<0.001
Hypertension	48,309 (16.3%)	15,775 (18.4%)	32,534 (15.5%)	<0.001
Diabetes	20,726 (7.0%)	7381 (8.6%)	13,345 (6.3%)	<0.001
Depression	32,883 (11.1%)	6260 (7.3%)	26,623 (12.7%)	<0.001
Anxiety	43,634 (14.8%)	8287 (9.7%)	35,347 (16.8%)	<0.001
Bipolar	17,145 (5.8%)	2402 (2.8%)	14,743 (7.0%)	<0.001
Stimulant use disorder	23,957 (8.1%)	2573 (3.0%)	21,384 (10.2%)	<0.001
Other psychoactive	27,012 (9.1%)	2431 (2.8%)	24,581 (11.7%)	<0.001
Sedative use	3408 (1.2%)	383 (0.4%)	3025 (1.4%)	<0.001
Other substance use disorder	48,853 (16.5%)	4887 (5.7%)	43,966 (20.9%)	<0.001
Opioid overdose	9176 (3.1%)	525 (0.6%)	8651 (4.1%)	<0.001
Opioid abuse	18,574 (6.3%)	1232 (1.4%)	17,342 (8.3%)	<0.001
Opioid dependence	34,605 (11.7%)	1999 (2.3%)	32,606 (15.5%)	<0.001

The index date was defined as the initial fentanyl exposure, either the date of the first fentanyl medication record or the first positive UDT result, whichever occurred first.

Data are No. (%), mean (SD), or median (IQR). SVI = social vulnerability index, Avg UDT = the mean of quantitative UDT values.

a Mann-Whitney U tests (two-sided) were performed to compare the medical- and illicit-source cohorts.

b Conditions were assessed within three months prior to the index date.

The illicit-source cohort was younger on average than the medical-source cohort (mean age, 42.0 versus 46.1 years; *P* < 0.001). A greater proportion of patients in the medical-source cohort were aged 50 years or older (43.9% versus 30.2%; Supplementary Fig. S1d). The illicit-source cohort was less commonly married (14.3% versus 26.3%; *P* < 0.001) and had a slightly higher SVI overall score (0.683 versus 0.677; *P* < 0.001). For state-level distributions (Fig. 1d), medical-source exposure was most common in North Carolina (10.6%), Ohio (10.5%), and California (10.3%), whereas illicit-source exposure was highly concentrated in Ohio (12.2%), followed by Massachusetts (10.5%) and California (8.8%).

Notably, patients in the medical-source cohort had a substantially higher mean number of fentanyl medication records (32.7 versus 7.58; *P* < 0.001) and lower UDT values (47.5 ng/mL versus 87.4 ng/mL; *P* < 0.001). Chronic diseases, including cancer, cardiovascular disease, hypertension, and diabetes, were more prevalent in the medical-source cohort, with cancer rates two-fold higher. In contrast, mental health disorders, substance use disorders, and opioid-related harmful outcomes, such as opioid overdose (nonfatal), abuse, and dependence, were more common in the illicit-source cohort. Rates were more than three-fold higher for stimulant use disorder and six-, five-, and six-fold higher for overdose, abuse, and dependence, respectively, compared with the medical-source cohort. All clinical conditions are presented in Supplementary Table S4, and region-stratified statistics for the medical-source cohort are provided in Supplementary Tables S6 and S7.

### Region-stratified subgroups in the illicit-source fentanyl exposure cohort

Among the 210,193 patients with inferred illicit-source fentanyl exposure (Table 2), the largest proportion resided in the Northeast (32.2%), followed by the South (30.2%), the Midwest (21.3%), and the West (16.1%). The Midwest had the highest mean number of fentanyl medication records (9.59) and the highest Avg UDT (136 ng/mL). The West had the lowest number of medication records (5.74) but the second highest Avg UDT (104 ng/mL). The Northeast showed relatively higher UDT testing frequency (mean count 4.87).

**Table 2:** Summary statistics on the illicit-source fentanyl exposure cohort, stratified by regions.

Variables	Total	West	Midwest	South	Northeast	*P* value^a^
Demographics						
Patients, No. (%)	210,193	33,760 (16.1%)	44,857 (21.3%)	63,475 (30.2%)	67,775 (32.2%)	NA
Male, No. (%)	124,944 (59.4%)	20,699 (61.3%)	25,228 (56.2%)	36,794 (58.0%)	42,004 (62.0%)	<0.001
Female, No. (%)	85,249 (40.6%)	13,061 (38.7%)	19,629 (43.8%)	26,681 (42.0%)	25,771 (38.0%)	<0.001
Married, No. (%)	29,961 (14.3%)	4946 (14.7%)	6405 (14.3%)	10,420 (16.4%)	8182 (12.1%)	<0.001
Age, mean (SD)	41.99 (15.168)	41.688 (16.115)	40.47 (14.597)	42.37 (15.698)	42.888 (14.353)	<0.001
Age, median (IQR)	39.0 (31.0, 53.0)	38.0 (30.0, 53.0)	38.0 (30.0, 50.0)	40.0 (31.0, 53.0)	41.0 (32.0, 53.0)	NA
Race, No. (%)						
White	129,945 (61.8%)	20,721 (61.4%)	28,751 (64.1%)	36,461 (57.4%)	43,918 (64.8%)	NA
Black or African American	45,383 (21.6%)	3832 (11.4%)	10,614 (23.7%)	18,959 (29.9%)	11,944 (17.6%)	NA
AIAN	6583 (3.1%)	2744 (8.1%)	1721 (3.8%)	683 (1.1%)	1430 (2.1%)	NA
Asian	1981 (0.9%)	830 (2.5%)	306 (0.7%)	325 (0.5%)	520 (0.8%)	NA
NHPI	706 (0.3%)	190 (0.6%)	115 (0.3%)	111 (0.2%)	289 (0.4%)	NA
Other race	10,445 (5.0%)	3031 (9.0%)	526 (1.2%)	2329 (3.7%)	4547 (6.7%)	NA
SVI, mean (SD)						
Overall	0.683 (0.27)	0.722 (0.242)	0.662 (0.279)	0.726 (0.233)	0.641 (0.297)	<0.001
Socioeconomic	0.635 (0.288)	0.65 (0.272)	0.624 (0.301)	0.698 (0.246)	0.58 (0.308)	<0.001
Household characteristics	0.608 (0.291)	0.572 (0.292)	0.621 (0.295)	0.648 (0.268)	0.58 (0.301)	<0.001
Housing type transportation	0.697 (0.248)	0.761 (0.235)	0.677 (0.232)	0.685 (0.248)	0.693 (0.256)	<0.001
Racial ethnic minority	0.695 (0.239)	0.804 (0.162)	0.618 (0.254)	0.72 (0.219)	0.672 (0.25)	<0.001
Fentanyl						
Medication record count, mean (SD)	7.577 (29.625)	5.739 (24.085)	9.585 (33.697)	7.129 (23.789)	7.615 (33.858)	<0.001
UDT count, mean (SD)	2.897 (7.026)	1.986 (2.632)	2.186 (3.359)	1.786 (2.921)	4.868 (11.331)	<0.001
Avg UDT value, mean (SD)	87.428 (112.875)	104.517 (126.956)	136.315 (163.416)	63.979 (76.402)	76.386 (103.092)	<0.001
Clinical conditions^b^						
Cancer, No. (%)	3327 (1.6%)	536 (1.6%)	727 (1.6%)	1032 (1.6%)	1031 (1.5%)	0.42
Cardiovascular disease	30,049 (14.3%)	4550 (13.5%)	6578 (14.7%)	9781 (15.4%)	9124 (13.5%)	<0.001
Hypertension	32,534 (15.5%)	4305 (12.8%)	7066 (15.8%)	11,060 (17.4%)	10,095 (14.9%)	<0.001
Diabetes	13,345 (6.3%)	1966 (5.8%)	2736 (6.1%)	4309 (6.8%)	4333 (6.4%)	<0.001
Depression	26,623 (12.7%)	2942 (8.7%)	6011 (13.4%)	6688 (10.5%)	10,976 (16.2%)	<0.001
Anxiety	35,347 (16.8%)	4144 (12.3%)	7797 (17.4%)	8713 (13.7%)	14,683 (21.7%)	<0.001
Bipolar	14,743 (7.0%)	1541 (4.6%)	3478 (7.8%)	3891 (6.1%)	5828 (8.6%)	<0.001
Stimulant use disorder	21,384 (10.2%)	3703 (11.0%)	4369 (9.7%)	5662 (8.9%)	7629 (11.3%)	<0.001
Other psychoactive	24,581 (11.7%)	2395 (7.1%)	5359 (11.9%)	6369 (10.0%)	10,444 (15.4%)	<0.001
Sedative use	3025 (1.4%)	318 (0.9%)	564 (1.3%)	826 (1.3%)	1312 (1.9%)	<0.001
Other substance use disorder	43,966 (20.9%)	6110 (18.1%)	9113 (20.3%)	10,980 (17.3%)	17,728 (26.2%)	<0.001
Opioid overdose	8651 (4.1%)	1096 (3.2%)	2016 (4.5%)	2556 (4.0%)	2975 (4.4%)	<0.001
Opioid abuse	17,342 (8.3%)	2340 (6.9%)	3714 (8.3%)	3797 (6.0%)	7475 (11.0%)	<0.001
Opioid dependence	32,606 (15.5%)	4262 (12.6%)	6171 (13.8%)	5456 (8.6%)	16,699 (24.6%)	<0.001

Three hundred twenty-six samples with missing state information were excluded. The index date was defined as the initial fentanyl exposure, either the date of the first fentanyl medication record or the first positive UDT result, whichever occurred first.

Data are No. (%), mean (SD), or median (IQR). AIAN = American Indian or Alaska Native, NHPI = Native Hawaiian or Other Pacific Islander, SVI = social vulnerability index, UDT = Urine drug testing, Avg UDT = the mean of quantitative UDT values.

a Kruskal-Wallis tests were performed to compare all regions.

b Conditions were assessed within three months prior to the index date.

The Northeast had the highest prevalence of mental health and substance use disorders and most opioid-related harmful outcomes, except for opioid overdose. In contrast, the South showed the lowest prevalence of opioid abuse (11.0% versus 6.0%; *P* < 0.001) and dependence (24.6% versus 8.6%; *P* < 0.001). All conditions are presented in Supplementary Table S5.

### Impact of illicit-source fentanyl exposure

Illicit-source fentanyl initiation was associated with increased risk of 30-day opioid-related harmful outcomes, including nonfatal opioid overdose, opioid abuse, and opioid dependence. In the entire cohort (Table 3), adjusted HRs for illicit-source initiation were 2.99 (95% confidence interval [CI], 2.71–3.29; *P* < 0.001) for opioid overdose, 1.96 (95% CI, 1.84–2.10; *P* < 0.001) for abuse, and 3.05 (95% CI, 2.89–3.22; *P* < 0.001) for dependence, compared with medical-source initiation, after confounding adjustment.

**Table 3:** Adjusted associations of illicit-source versus medical-source fentanyl initiation with opioid-related harmful outcomes in the entire cohort and subgroups stratified by substance use disorders, mental health conditions, and chronic conditions.

Subgroup	# Patients	Overdose	Abuse	Dependence
All	194,348	2.99 (2.71–3.29); <0.001	1.96 (1.84–2.10); <0.001	3.05 (2.89–3.22); <0.001
Substance use disorder				
Positive	77,499	3.00 (2.62–3.44); <0.001	2.07 (1.88–2.27); <0.001	3.32 (3.07–3.59); <0.001
Negative	116,849	3.04 (2.66–3.48); <0.001	2.08 (1.89–2.29); <0.001	3.17 (2.94–3.41); <0.001
Mental health conditions				
Positive	64,029	2.80 (2.38–3.29); <0.001	2.49 (2.21–2.80); <0.001	3.26 (2.96–3.58); <0.001
Negative	130,319	3.09 (2.75–3.48); <0.001	1.78 (1.64–1.92); <0.001	2.97 (2.78–3.17); <0.001
Chronic conditions				
Positive	117,891	3.54 (3.11–4.02); <0.001	2.47 (2.26–2.71); <0.001	3.50 (3.25–3.76); <0.001
Negative	76,457	2.54 (2.18–2.96); <0.001	1.58 (1.43–1.75); <0.001	2.79 (2.57–3.03); <0.001

Adjusted hazard ratios (HRs) with 95% confidence intervals and *P* values are presented for nonfatal opioid overdose, opioid abuse, and opioid dependence, with medical-source fentanyl initiation as the reference group.

Positive subgroups included patients with prior corresponding conditions, whereas negative subgroups included those without. Patients with any prior opioid-related harmful outcomes were excluded. No unbalanced covariates remained after stabilized inverse probability weighting in any analysis.

In analyses restricted to the illicit-source cohort alone, associations remained significant, with adjusted HRs of 2.84 (95% CI, 2.46–3.28; *P* < 0.001) for opioid overdose, 1.53 (95% CI, 1.40–1.68; *P* < 0.001) for abuse, and 2.25 (95% CI, 2.09–2.42; *P* < 0.001) for dependence.

Across all clinical subgroups in the entire cohort (Table 3), illicit-source initiation was consistently associated with increased risk of 30-day opioid-related harmful outcomes. For nonfatal opioid overdose, adjusted HRs were 3.00 versus 3.04 for patient subgroups with and without substance use disorders, respectively, and 2.80 versus 3.09 (mental health disorders), and 3.54 versus 2.54 (chronic conditions). The largest differences in HRs were observed for chronic conditions, with consistently higher HRs among patients with chronic conditions than among those without. Similar findings were observed in subgroup analyses restricted to the illicit-source cohort alone (Supplementary Table S8), in which illicit-source initiation remained consistently associated with increased risk of these outcomes across clinical subgroups.

Sensitivity analyses using alternative UDT screening windows demonstrated consistent findings (Supplementary Table S9). Increased risks were also observed across alternative follow-up outcome windows, with decreasing HRs as the prediction window lengthened (Supplementary Table S10). Covariate balance (SMDs) is presented in Supplementary Tables S11 and S12 and Supplementary Fig. S2, and details of the propensity score model are provided in Supplementary Tables S13 and S14 and Supplementary Fig. S3.

### Temporal patterns in opioid-related harmful outcomes

**Fig. 2:**
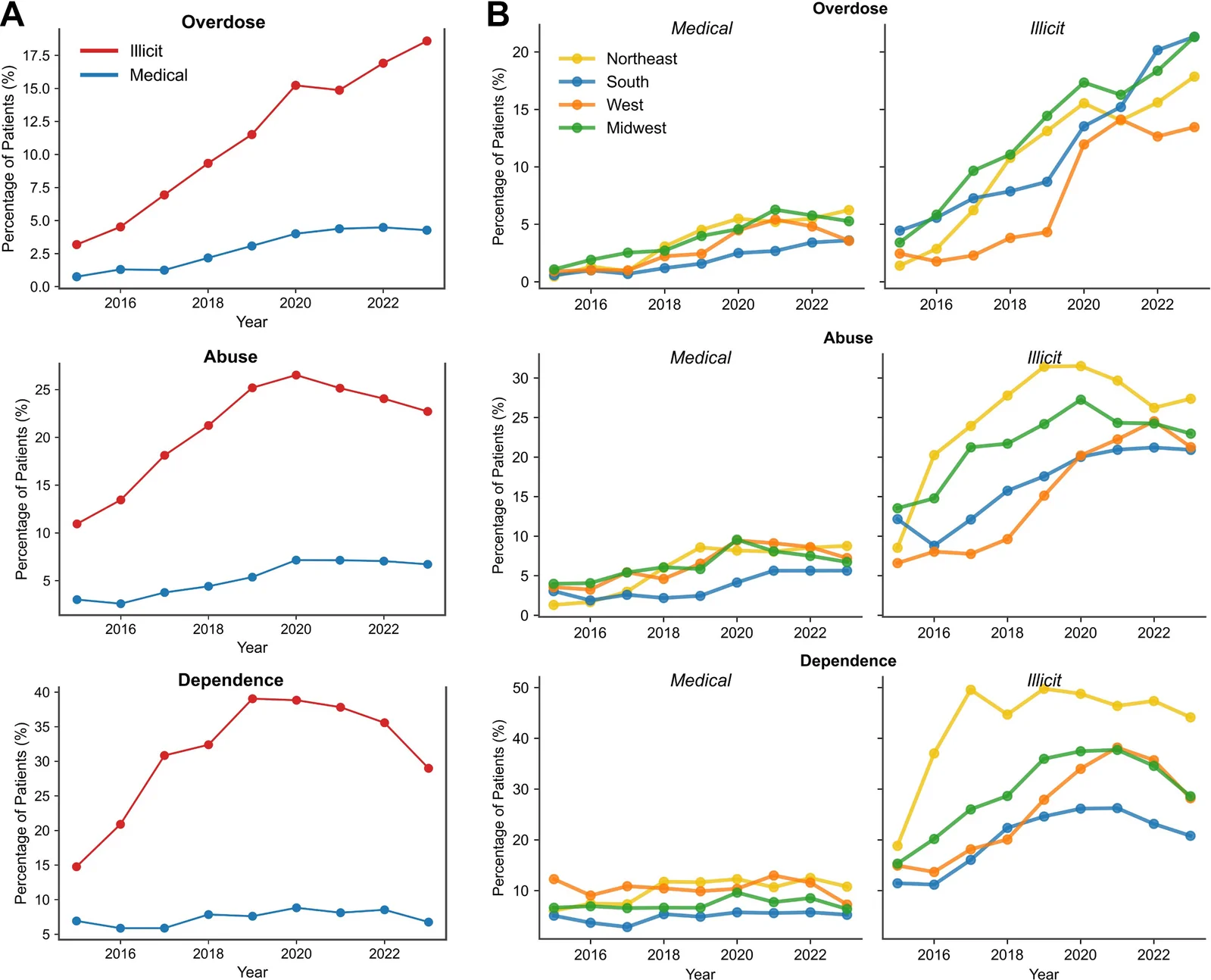
**Temporal patterns in opioid overdose (nonfatal), dependence, and abuse from 2015 to 2023.** A. the medical- and illicit-source fentanyl exposure cohorts; and B. the four regions within medical-source (left) and illicit-source fentanyl exposure cohorts (right). Medical = medical-source fentanyl exposure cohort, Illicit = illicit-source fentanyl exposure cohort.

From 2015 to 2023, nonfatal opioid overdose prevalence was consistently higher in the illicit-source fentanyl exposure cohort (Fig. 2a). Overdose prevalence in the illicit-source cohort increased markedly over time, reaching 18.6%, whereas only a modest increase was observed in the medical-source cohort, reaching 4.3%. For opioid dependence and abuse, the illicit-source cohort exhibited substantially higher prevalence and steeper year-over-year increases than the medical-source cohort until 2020. After 2020, prevalence in the illicit-source cohort declined, particularly for dependence, but remained consistently higher than in the medical-source cohort throughout the study period.

Region-stratified temporal patterns followed the overall cohort-level patterns (Fig. 2b). Within the medical-source cohort, regional trajectories were relatively stable and comparable over time. In contrast, the illicit-source cohort showed regional variation. The Northeast generally exhibited higher prevalence levels of opioid abuse and dependence, with sharp increases beginning in 2016 and peaking around 2019. Across all regions, prevalence remained consistently higher in the illicit-source cohort than in the medical-source cohort throughout the study period.

## Discussion

In this retrospective cohort study, we inferred medical- and illicit-source fentanyl exposure by linking UDT results with medication records from one of the largest U.S. EHR databases using a sequential time-window screening method. We found that illicit-source fentanyl exposure was associated with a sustained increase in nonfatal opioid overdoses over the study period, together with substantially greater clinical burden and risks of opioid-related harmful outcomes than medical-source fentanyl exposure. We further observed distinct demographic and clinical characteristics between exposure groups and elevated risks associated with illicit-source fentanyl initiation across diverse clinical subgroups. These findings are consistent with the evolving landscape of the opioid overdose crisis, in which illicitly manufactured fentanyl has emerged as a major contributor.^11^ Together, these findings provide one of the first national-scale assessments of the clinical impact of illicit-source fentanyl exposure in the U.S. using individual-level EHR data and highlight the need to distinguish these two populations for surveillance, risk stratification, and targeted interventions.

Fentanyl remains an important synthetic opioid for managing severe pain.^4^^,^^5^ Although highly effective, its high potency also increases the risk of misuse and overdose.^7^^,^^26^^,^^27^ In recent years, fentanyl has become a major driver of the opioid overdose crisis in the U.S.,^13^ largely due to its increasing presence in the illicit drug market.^10^^,^^11^ This shift has prompted significant public health responses, including expanded distribution of naloxone,^28^ enhanced drug monitoring, and stricter regulatory measures to prevent the spread of fentanyl-related substances. A major challenge in addressing the overdose epidemic is the lack of comprehensive data-driven studies on illicit fentanyl use, as a significant proportion of overdose cases involve fentanyl obtained outside of formal healthcare or prescription systems.^11^^,^^12^ Previous studies have used UDT data to track nonprescribed fentanyl co-detection with other substances,^18^^,^^19^ yet they have provided limited insight into how the source of fentanyl exposure influences opioid-related harmful outcomes. This study addresses this gap by differentiating medical- and potential illicit-source fentanyl exposure at the individual level, enabling direct comparison of longitudinal patterns and associated clinical risks by source of fentanyl exposure.

Marked differences were observed between the medical- and illicit-source fentanyl exposure cohorts. Individuals in the medical-source cohort were older and had a higher burden of chronic conditions, such as cancer, cardiovascular disease, diabetes, and hypertension,^29^ diseases consistent with legitimate therapeutic indications for fentanyl use. This clinical profile was further reflected by a substantially higher number of fentanyl medication records. In contrast, individuals in the illicit-source cohort were younger,^30^ less likely to be married, and had a markedly higher prevalence of mental health disorders, substance use disorders, and opioid-related harmful outcomes. This greater clinical burden may reflect differences in exposure context, including uncontrolled dosing, increased likelihood of polysubstance use, and lack of medical supervision. These findings highlight the distinct demographic and clinical differences between the medical-source and illicit-source fentanyl exposure populations.

A particularly noteworthy finding was the dynamic temporal pattern in nonfatal opioid overdoses driven by medical-versus potential illicit-source fentanyl exposures. Overdose cases driven by medical-source fentanyl increased modestly over time, whereas the illicit-source cohort showed a sustained increase through 2023. These patterns indicate that illicit-source fentanyl remains a significant and growing threat to public health nationwide. This shift in overdose driving force underscores the critical impact of policy changes, such as opioid prescription restrictions, on reducing medical-driven fentanyl misuse. However, the continued rise in overdoses linked to illicit fentanyl highlights a persistent and escalating crisis that requires urgent attention.

Furthermore, illicit-source fentanyl initiation was associated with increased short-term risk of opioid-related harmful outcomes compared with medical-source initiation. Individuals initiating fentanyl from illicit sources experienced approximately a two-fold higher risk of opioid abuse, and three-fold higher risks of opioid overdose and dependence within 30 days, with elevated risks also observed over extended follow-up periods (∼180 days). These associations were also observed in analyses restricted to the illicit-source cohort, suggesting that the increased risks are not solely explained by differences between exposure groups, but also reflect the importance of the source of initiation. Across all clinical subgroups, illicit-source initiation was associated with increased risk of outcomes, regardless of the underlying clinical conditions. These findings suggest that the adverse impact of illicit-source fentanyl initiation extends across a broad range of clinical populations and is not limited to specific high-risk groups.

This study has several limitations. First, illicit fentanyl exposure was inferred from fentanyl UDT results. Patients without UDT records were excluded from the current analysis, which may have led to an incomplete assessment of the impact of illicit fentanyl exposure. In addition, UDT-based exposure classification may be subject to misclassification, which could lead to underestimation of the true association. However, UDT provides an objective measure of drug exposure, offering greater reliability than self-reported or survey-based data. Importantly, compared with previous studies that had smaller sample sizes and cross-sectional designs, this study is one of the first to estimate the impact of illicit fentanyl use across all U.S. states over an extended period. The patterns observed in this study are also supported by previous studies.^11^^,^^12^^,^^30^ Second, although substance use disorders were included in the analyses to account for potential polysubstance use, they may not fully capture all concurrent substance use. Third, patients classified as the medical-source may have been exposed to illicit fentanyl between the time of medical administration and the UDT. However, given that these patients were likely from inpatient settings and the time window between medical fentanyl use and testing was short, the likelihood of illicit exposure in this group is low. Fourth, we were unable to perform a comparative analysis for fentanyl-associated events at the state level due to small sample sizes in several states. Instead, we conducted analyses based on major U.S. regions, which still provided valuable insights into regional variations in opioid-related harmful outcomes. Fifth, fatal opioid overdose could not be examined because cause-of-death information was not available in Epic Cosmos. Sixth, healthcare encounters occurring outside Epic Cosmos may not be captured, which may limit the generalizability of our findings. However, because Epic Cosmos is broadly representative of the U.S. population seeking healthcare, the potential impact on our findings is likely to be limited. Seventh, missingness in UDT data was primarily due to incomplete laboratory information in UDT records, such as missing specimen collection dates or laboratory values. This suggests that missingness was likely related to data completeness; however, we were unable to assess whether it could be considered missing at random. The potential impact of missing data on the study findings therefore cannot be completely excluded. Finally, the COVID-19 pandemic may have influenced healthcare utilization, access to care, substance use patterns, and clinical outcomes. However, the potential impact of the pandemic was not explicitly evaluated in the present study.

In summary, this cohort study represents one of the first and largest observational studies to estimate the scale and impact of illicit-source fentanyl exposure on the national level and among major geographic regions in the U.S. The evolving dynamics of fentanyl-driven harmful outcomes across U.S. healthcare systems suggest that illicit-source fentanyl use continues to play a major role in the ongoing overdose epidemic, even as harms associated with medical-source fentanyl use remain comparatively modest. Our findings underscore the complexity of the underlying mechanisms contributing to the opioid-associated public health crisis and highlight the need for a deeper understanding of the interactions between medical and illicit fentanyl use. Future research should focus on characterizing high-risk populations who transition from medical-to illicit-source fentanyl use to better inform targeted prevention and intervention strategies. In addition, analyses of healthcare utilization, care quality, and drug market dynamics were not addressed in this study and represent important directions for future research.

## Contributors

WS and PZ conceptualized and supervised this study. SL and WS designed the longitudinal cohort study, and PZ processed and verified the dataset and methods underlying this study. SL conducted the data and statistical analysis. SL, WS, and PZ wrote the original draft, with critical contributions from DWB and RDU. DWB and RDU provided important clinical guidance. All authors assisted with the literature search, data analysis, and data interpretation. SL and PZ accessed and verified the underlying data. All authors read and approved the final manuscript and had final responsibility for the decision to submit for publication.

## Data sharing statement

The data we used is from the Epic Cosmos. Data cannot be downloaded or removed from Cosmos by individual users, but any participating healthcare organization can grant data access to users.

## Editor note

The Lancet Group takes a neutral position with respect to territorial claims in published maps and institutional affiliations.

## Declaration of interests

DWB reports personal fees from Relyens, personal fees and equity from AESOP, personal fees and equity from FeelBetter, and personal fees and equity from Guided Clinical Solutions, outside the submitted work. RDU reports grants from National Institutes of Health, outside the submitted work. PZ reports grants from National Institute of Allergy and Infectious Diseases, National Institute of General Medical Sciences, National Cancer Institute, and National Library of Medicine, outside the submitted work. The other authors declare no competing interests.
