# Supplementary material for: Longitudinal patterns of fentanyl utilisation (medical versus illicit sources) in the United States 2015–2023: a retrospective cohort study
Source: eClinicalMedicine. 2026 Aug 14;99:104141. doi: 10.1016/j.eclinm.2026.104141 (PMC13499189; doi:10.1016/j.eclinm.2026.104141)
Supplement: Supplementary file 1 [file mmc1.pdf]

Supplementary Material

Lee S, Song W, Bates DW, Urman RD, Zhang P. Longitudinal patterns of fentanyl utilisation (medical vs illicit sources) across the United States 2015-2023: a retrospective cohort study

Table of Contents

Data source ***2***

Study cohort ***4***

- **Statistics** **4**
- **Study cohort** **5**
- **Illicit-source fentanyl exposure cohort** **6**
- **Medical-source fentanyl exposure cohort** **7**

Statistical Analysis ***9***

- **Subgroup analysis** **9**
- **Sensitivity Analysis** **10**
- **Evaluation of baseline covariate balance** **12**
- **Model Specifications** **15**

Reference ***18***

Data source

Supplementary Table 1. Geographic region of the United States.[1]

Regions	States
West	Alaska, Arizona, California, Colorado, Hawaii, Idaho, Montana, Nevada, New Mexico, Oregon, Utah, Washington, Wyoming.
Midwest	Illinois, Indiana, Iowa, Kansas, Michigan, Minnesota, Missouri, Nebraska, North Dakota, Ohio, South Dakota, Wisconsin
South	Alabama, Arkansas, Delaware, District of Columbia, Florida, Georgia, Kentucky, Louisiana, Maryland, Mississippi, North Carolina, Oklahoma, South Carolina, Tennessee, Texas, Virginia, West Virginia
Northeast	Connecticut, Maine, Massachusetts, New Hampshire, New Jersey, New York, Pennsylvania, Rhode Island, Vermont

Supplementary Table 2. International classification of diseases codes (ICD)-10 to identify clinical conditions.[2–5, 7, 8]

	**Diseases**	**ICD-10**
**Chronic diseases**	Cancer	C00-C97
	Tuberculosis	A15–A19
	Herpes zoster	B02
	Human immunodeficiency virus	B20–B24
	Thyroidal diseases	E00–E07
	Diabetes	E10–E14
	Metabolic diseases	E65–E68
	Hyperlipidaemia	E78
	Epilepsy	G40
	Cardiovascular disease	I00–I09, I16–I99
	Hypertension	I10–I15
	Chronic obstructive pulmonary disease	J44
	Asthma	J45–J46
	Bronchiectasis	J47
	Gastro-oesophageal reflux	K21
	Psoriasis	L40
	Rheumatoid arthritis	M05–M14
	Osteoarthritis	M15–M19
	Ankylosing spondylitis	M45
	Chronic back pain	M53–M54
	Fibromyalgia/myalgia	M79
	Osteoporosis	M80–M82
	Other chronic musculoskeletal problems	M00–M03, M20–M43, M46–M51, M60–M77, M83–M99
	Renal disease	N18–N19
**Mental health disorders**	ADHD	F90
	Depression	F32, F33
	Anxiety	F41, F42, F43, F44, F45, F48
	Bipolar	F30, F31, F34, F39
	Schizophrenia/Psychoses	F20, F21, F22, F23, F24, F25, F28, F29
**Opioid-related harmful outcomes**	Opioid overdose	T400-T404, T406 (excluding adverse effect and underdosing)
	Opioid dependence	F11.2
	Opioid abuse	F11.1
**Substance use disorders**	Stimulant use disorder	F141, F142, F151, F152, R782, T405, T4360-T4363, T4369
	Cannabis use disorder	F12
	Hallucinogen use disorder	F161, F162
	Other psychoactive use	F191, F192
	Sedative use	F131, T423, T424, T426
	Alcohol use disorder	F10
	Tobacco use disorder	F17, Z720
	Other substance use disorder	F13, F14, F15, F16, F18, F19

Supplementary Table 3. Fentanyl medication records included in the analysis.

Records	Routes
**Order**	Transdermal, oral, buccal, sublingual, nasal, topical, intravenous (including bolus and drip), intramuscular, subcutaneous, epidural, intrathecal, intraspinal, injection, parenteral, intradermal, infiltration, intratympanic, oropharyngeal, respiratory (inhalation), and other or unspecified routes.
**Administration**	Intravenous (including bolus and drip), epidural, intrathecal, intramuscular, subcutaneous, transdermal, buccal, nasal, topical, infiltration, intraspinal, injection, respiratory (inhalation), oropharyngeal, parenteral, and other or unknown routes.

Study cohort

Statistics

Supplementary Figure 1. Age distribution at the index date. A. The medical- and illicit-source fentanyl use cohorts; B. The distribution in males; C. The distribution in females; D. The age group distribution for the medical- and illicit-source cohorts. The index date=the fentanyl initiation date.

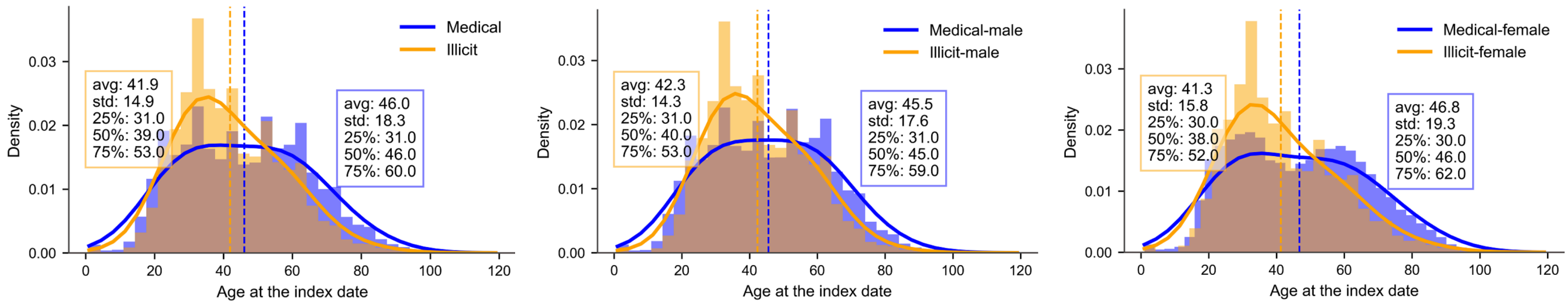

A **B** **C**

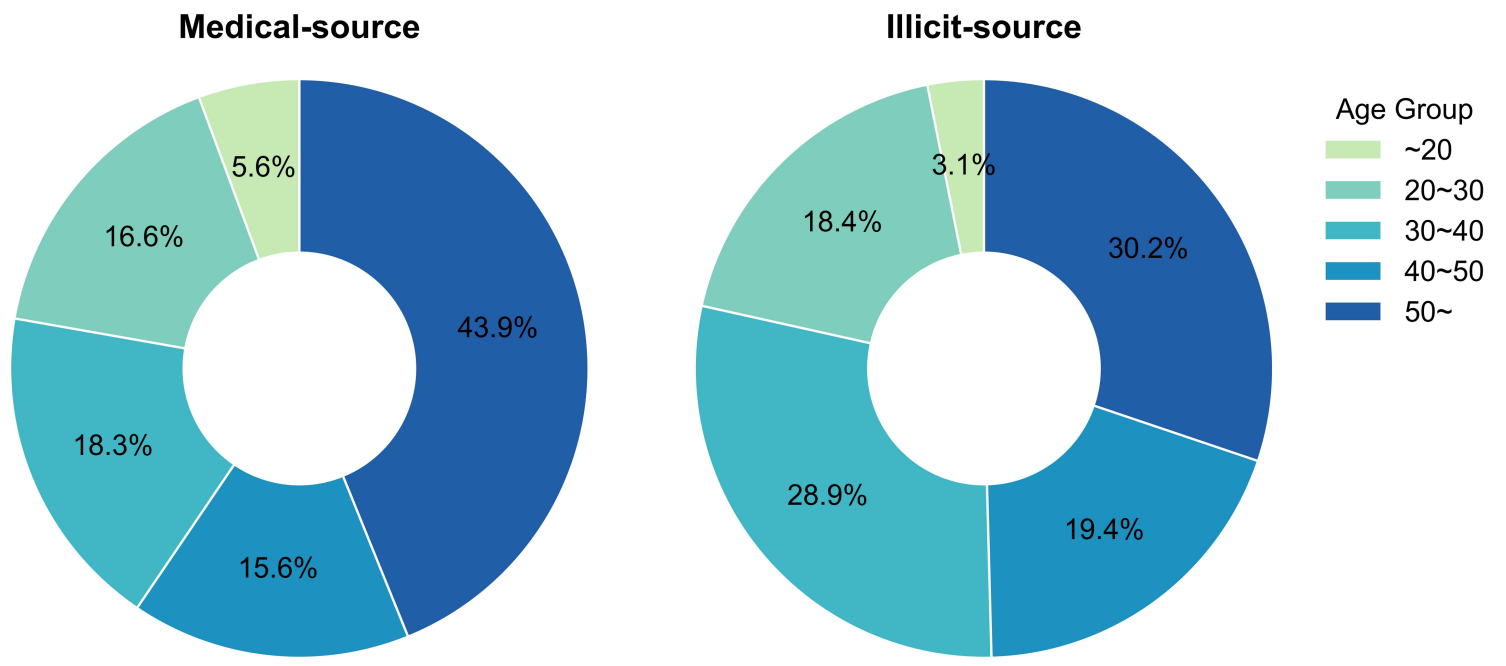

D

Study cohort

Supplementary Table 4. Summary statistics of diseases on the entire cohort, and the medical- and illicit-source cohorts.

Variables	Total	Medical-source	Illicit-source	*P* value[a]
Patients, No. (%)	295728	85535 (28.9%)	210193 (71.1%)	NA
Fentanyl medication record count, mean (SD)	14.85 (43.757)	32·723 (63.352)	7.577 (29.625)	<.001
Avg UDT value, mean (SD)	77.661 (101.559)	47·542 (61.187)	87.428 (112.875)	<.001
**Clinical Conditions[b]**				
ADHD, n (%)	5696 (1.9%)	807 (0.9%)	4889 (2.3%)	<.001
Alcohol disorder, n (%)	20311 (6.9%)	4417 (5.2%)	15894 (7.6%)	<.001
Ankylosing spondylitis, n (%)	156 (0.1%)	49 (0.1%)	107 (0.1%)	0.49
Asthma, n (%)	14934 (5.0%)	3486 (4.1%)	11448 (5.4%)	<.001
Bronchiectasis, n (%)	285 (0.1%)	88 (0.1%)	197 (0.1%)	0.47
Cannabis disorder, n (%)	13534 (4.6%)	2235 (2.6%)	11299 (5.4%)	<.001
Chronic back pain, n (%)	28195 (9.5%)	7348 (8.6%)	20847 (9.9%)	<.001
Chronic obstructive pulmonary disease, n (%)	11407 (3.9%)	3714 (4.3%)	7693 (3.7%)	<.001
Epilepsy, n (%)	5865 (2.0%)	1625 (1.9%)	4240 (2.0%)	0.038
Fibromyalgia/Myalgia, n (%)	24849 (8.4%)	5971 (7.0%)	18878 (9.0%)	<.001
Gastro-oesophageal reflux, n (%)	16976 (5.7%)	5352 (6.3%)	11624 (5.5%)	<.001
Hallucinogen use disorder, n (%)	569 (0.2%)	59 (0.1%)	510 (0.2%)	<.001
Herpes zoster, n (%)	417 (0.1%)	143 (0.2%)	274 (0.1%)	0.016
Human immunodeficiency virus, n (%)	2619 (0.9%)	461 (0.5%)	2158 (1.0%)	<.001
Hyperlipidaemia, n (%)	22749 (7.7%)	8697 (10.2%)	14052 (6.7%)	<.001
Metabolic diseases, n (%)	16436 (5.6%)	5314 (6.2%)	11122 (5.3%)	<.001
Osteoarthritis, n (%)	10798 (3.7%)	3465 (4.1%)	7333 (3.5%)	<.001
Osteoporosis, n (%)	1850 (0.6%)	778 (0.9%)	1072 (0.5%)	<.001
Other chronic musculoskeletal problems, n (%)	44184 (14.9%)	12067 (14.1%)	32117 (15.3%)	<.001
Psoriasis, n (%)	825 (0.3%)	258 (0.3%)	567 (0.3%)	0.14
Renal disease, n (%)	7626 (2.6%)	2858 (3.3%)	4768 (2.3%)	<.001
Rheumatoid arthritis, n (%)	4132 (1.4%)	1427 (1.7%)	2705 (1.3%)	<.001
Schizophrenia, n (%)	10242 (3.5%)	1253 (1.5%)	8989 (4.3%)	<.001
Thyroid, n (%)	9323 (3.2%)	3092 (3.6%)	6231 (3.0%)	<.001
Tobacco disorder, n (%)	67303 (22.8%)	12666 (14.8%)	54637 (26.0%)	<.001
Tuberculosis, n (%)	129 (0.0%)	32 (0.0%)	97 (0.0%)	0.30

Data are No. (%) or mean (SD). Avg UDT=the mean of quantitative urine drug testing values.
[a]Mann-Whitney U tests (two-sided) were performed to compare the medical- and illicit-source fentanyl use cohorts
[b]Conditions were assessed within three months prior the index date.

Illicit-source fentanyl exposure cohort

Supplementary Table 5. Summary statistics of diseases on the illicit-source cohort, stratified by regions.

Variables	Total	West	Midwest	South	Northeast	*P* value[a]
Patients, No. (%)	210193	33760 (16.1%)	44857 (21.3%)	63475 (30.2%)	67775 (32.2%)	NA
Fentanyl medication record count, mean (SD)	7.577 (29.625)	5.739 (24.085)	9.585 (33.697)	7.129 (23.789)	7.615 (33.858)	<.001
Avg UDT value, mean (SD)	87.428 (112.875)	104.517 (126.956)	136.315 (163.416)	63.979 (76.402)	76.386 (103.092)	<.001
**Clinical conditions[b]**						
ADHD, n (%)	4889 (2.3%)	406 (1.2%)	1084 (2.4%)	1044 (1.6%)	2354 (3.5%)	<.001
Alcohol disorder, n (%)	15894 (7.6%)	1959 (5.8%)	3223 (7.2%)	4039 (6.4%)	6669 (9.8%)	<.001
Ankylosing spondylitis, n (%)	107 (0.1%)	27 (0.1%)	25 (0.1%)	26 (0.0%)	29 (0.0%)	0.048
Asthma, n (%)	11448 (5.4%)	1376 (4.1%)	2652 (5.9%)	2828 (4.5%)	4589 (6.8%)	<.001
Bronchiectasis, n (%)	197 (0.1%)	35 (0.1%)	43 (0.1%)	63 (0.1%)	56 (0.1%)	0.69
Cannabis disorder, n (%)	11299 (5.4%)	1355 (4.0%)	2854 (6.4%)	3217 (5.1%)	3868 (5.7%)	<.001
Chronic back pain, n (%)	20847 (9.9%)	3060 (9.1%)	5024 (11.2%)	5943 (9.4%)	6810 (10.0%)	<.001
Chronic obstructive pulmonary disease, n (%)	7693 (3.7%)	882 (2.6%)	1981 (4.4%)	2449 (3.9%)	2379 (3.5%)	<.001
Epilepsy, n (%)	4240 (2.0%)	582 (1.7%)	879 (2.0%)	1450 (2.3%)	1327 (2.0%)	<.001
Fibromyalgia/Myalgia, n (%)	18878 (9.0%)	2930 (8.7%)	4553 (10.2%)	5057 (8.0%)	6332 (9.3%)	<.001
Gastro-oesophageal reflux, n (%)	11624 (5.5%)	1319 (3.9%)	2759 (6.2%)	3667 (5.8%)	3877 (5.7%)	<.001
Hallucinogen use disorder, n (%)	510 (0.2%)	41 (0.1%)	85 (0.2%)	81 (0.1%)	303 (0.4%)	<.001
Herpes zoster, n (%)	274 (0.1%)	53 (0.2%)	57 (0.1%)	76 (0.1%)	88 (0.1%)	0.49
Human immunodeficiency virus, n (%)	2158 (1.0%)	362 (1.1%)	296 (0.7%)	708 (1.1%)	791 (1.2%)	<.001
Hyperlipidaemia, n (%)	14052 (6.7%)	1693 (5.0%)	3016 (6.7%)	4518 (7.1%)	4822 (7.1%)	<.001
Metabolic diseases, n (%)	11122 (5.3%)	1502 (4.4%)	2761 (6.2%)	3449 (5.4%)	3405 (5.0%)	<.001
Osteoarthritis, n (%)	7333 (3.5%)	1048 (3.1%)	1794 (4.0%)	2163 (3.4%)	2327 (3.4%)	<.001
Osteoporosis, n (%)	1072 (0.5%)	226 (0.7%)	207 (0.5%)	330 (0.5%)	308 (0.5%)	<.001
Other chronic musculoskeletal problems, n (%)	32117 (15.3%)	5212 (15.4%)	7471 (16.7%)	8900 (14.0%)	10524 (15.5%)	<.001
Psoriasis, n (%)	567 (0.3%)	64 (0.2%)	112 (0.2%)	149 (0.2%)	241 (0.4%)	<.001
Renal disease, n (%)	4768 (2.3%)	671 (2.0%)	960 (2.1%)	1781 (2.8%)	1356 (2.0%)	<.001
Rheumatoid arthritis, n (%)	2705 (1.3%)	463 (1.4%)	582 (1.3%)	902 (1.4%)	757 (1.1%)	<.001
Schizophrenia, n (%)	8989 (4.3%)	1444 (4.3%)	1984 (4.4%)	2892 (4.6%)	2665 (3.9%)	<.001
Thyroid, n (%)	6231 (3.0%)	1015 (3.0%)	1314 (2.9%)	1863 (2.9%)	2039 (3.0%)	0.79
Tobacco disorder, n (%)	54637 (26.0%)	5947 (17.6%)	13389 (29.8%)	16247 (25.6%)	19035 (28.1%)	<.001
Tuberculosis, n (%)	97 (0.0%)	16 (0.0%)	21 (0.0%)	32 (0.1%)	28 (0.0%)	0.89

Data are No. (%) or mean (SD). Avg UDT=the mean of quantitative urine drug testing values.
[a]Kruskal-Wallis tests were performed to compare all region subcohorts.
[b]Conditions were assessed within three months prior the index date.

Medical-source fentanyl exposure cohort

Supplementary Table 6. Summary statistics on the medical-source fentanyl exposure cohort, stratified by regions.

Variables		Total	West	Midwest	South	Northeast	*P* value[a]
**Demographics**							
Patients, No. (%)		85535	15335 (17.9%)	18522 (21.7%)	34881 (40.8%)	16753 (19.6%)	NA
Male, No. (%)		51163 (59.8%)	9411 (61.4%)	11001 (59.4%)	20761 (59.5%)	9958 (59.4%)	0.0003
Married, No. (%)		22458 (26.3%)	3718 (24.2%)	4737 (25.6%)	9165 (26.3%)	4831 (28.8%)	<.001
Age, mean (SD)		46.079 (18.39)	45.388 (18.122)	45.849 (18.001)	45.184 (18.415)	48.824 (18.703)	<.001
Age, median (IQR)		46.0 (31.0, 60.0)	44.0 (31.0, 59.0)	45.0 (31.0, 60.0)	44.0 (30.0, 59.0)	50.0 (33.0, 62.0)	NA
Race, No. (%)	White	51283 (60.0%)	9587 (62.5%)	12235 (66.1%)	17898 (51.3%)	11552 (69.0%)	NA
	Black or African American	20393 (23.8%)	1610 (10.5%)	3848 (20.8%)	12171 (34.9%)	2759 (16.5%)	NA
	AIAN	2026 (2.4%)	741 (4.8%)	510 (2.8%)	539 (1.5%)	236 (1.4%)	NA
	Asian	1377 (1.6%)	597 (3.9%)	217 (1.2%)	310 (0.9%)	253 (1.5%)	NA
	NHPI	268 (0.3%)	89 (0.6%)	44 (0.2%)	93 (0.3%)	42 (0.3%)	NA
	Other Race	4073 (4.8%)	1357 (8.8%)	270 (1.5%)	1578 (4.5%)	864 (5.2%)	NA
SVI, mean (SD)	Overall	0.677 (0.272)	0.742 (0.247)	0.612 (0.29)	0.738 (0.232)	0.566 (0.296)	<.001
	Socioeconomic	0.635 (0.288)	0.68 (0.28)	0.567 (0.309)	0.712 (0.242)	0.511 (0.297)	<.001
	Household Characteristics	0.622 (0.282)	0.636 (0.291)	0.595 (0.288)	0.673 (0.258)	0.535 (0.289)	<.001
	Housing Type Transportation	0.672 (0.256)	0.744 (0.242)	0.642 (0.247)	0.678 (0.253)	0.631 (0.268)	<.001
	Racial Ethnic Minority	0.685 (0.246)	0.823 (0.157)	0.571 (0.254)	0.737 (0.211)	0.577 (0.27)	<.001
**Fentanyl**							
Medication record count, mean (SD)		32.723 (63.352)	30.974 (61.601)	37.687 (75.501)	28.351 (52.374)	37.971 (69.994)	<.001
UDT count, mean (SD)		1.405 (1.858)	1.401 (1.786)	1.398 (1.584)	1.227 (1.095)	1.786 (3.037)	<.001
Avg UDT value, mean (SD)		47.542 (61.187)	55.796 (75.08)	49.292 (66.327)	30.223 (36.17)	58.748 (74.195)	<.001
**Clinical Conditions**[b]							
Cancer, No. (%)		2732 (3.2%)	371 (2.4%)	564 (3.0%)	1013 (2.9%)	784 (4.7%)	<.001
Cardiovascular Disease		14660 (17.1%)	2158 (14.1%)	3509 (18.9%)	5852 (16.8%)	3138 (18.7%)	<.001
Hypertension		15775 (18.4%)	2220 (14.5%)	3833 (20.7%)	6464 (18.5%)	3257 (19.4%)	<.001
Diabetes		7381 (8.6%)	1182 (7.7%)	1784 (9.6%)	2896 (8.3%)	1519 (9.1%)	<.001
Depression		6260 (7.3%)	850 (5.5%)	1708 (9.2%)	2161 (6.2%)	1541 (9.2%)	<.001
Anxiety		8287 (9.7%)	1128 (7.4%)	2180 (11.8%)	2889 (8.3%)	2089 (12.5%)	<.001
Bipolar		2402 (2.8%)	350 (2.3%)	656 (3.5%)	827 (2.4%)	569 (3.4%)	<.001
Stimulant Use Disorder		2573 (3.0%)	720 (4.7%)	575 (3.1%)	878 (2.5%)	399 (2.4%)	<.001
Other Psychoactive		2431 (2.8%)	427 (2.8%)	651 (3.5%)	881 (2.5%)	472 (2.8%)	<.001
Sedative Use		383 (0.4%)	49 (0.3%)	86 (0.5%)	157 (0.5%)	90 (0.5%)	0.032
Other Substance Use Disorder		4887 (5.7%)	1116 (7.3%)	1162 (6.3%)	1717 (4.9%)	892 (5.3%)	<.001
Opioid Overdose		525 (0.6%)	79 (0.5%)	140 (0.8%)	171 (0.5%)	133 (0.8%)	<.001
Opioid Abuse		1232 (1.4%)	276 (1.8%)	303 (1.6%)	366 (1.0%)	287 (1.7%)	<.001
Opioid Dependence		1999 (2.3%)	442 (2.9%)	407 (2.2%)	544 (1.6%)	606 (3.6%)	<.001

Forty-four samples with missing state information were excluded. Data are No. (%), mean (SD), or median (IQR). AIAN=American Indian or Alaska Native, NHPI=Native Hawaiian or Other Pacific Islander, SVI=social vulnerability index, UDT=Urine drug testing, Avg UDT=the mean of quantitative UDT values.

[a]Kruskal-Wallis tests were performed to compare all region subcohorts.

[b]Conditions were assessed within three months prior the index date.

Supplementary Table 7. Summary statistics of diseases on the medical-source cohort, stratified by regions.

Variables	Total	West	Midwest	South	Northeast	*P* value[a]
Patients, No. (%)	85535	15335 (17.9%)	18522 (21.7%)	34881 (40.8%)	16753 (19.6%)	NA
Fentanyl medication record count, mean (SD)	32.723 (63.352)	30.974 (61.601)	37.687 (75.501)	28.351 (52.374)	37.971 (69.994)	<.001
Avg UDT value, mean (SD)	47.542 (61.187)	55.796 (75.08)	49.292 (66.327)	30.223 (36.17)	58.748 (74.195)	<.001
**Clinical conditions[b]**						
ADHD, n (%)	807 (0.9%)	85 (0.6%)	206 (1.1%)	297 (0.9%)	219 (1.3%)	<.001
Alcohol disorder, n (%)	4417 (5.2%)	774 (5.0%)	1175 (6.3%)	1486 (4.3%)	982 (5.9%)	<.001
Ankylosing spondylitis, n (%)	49 (0.1%)	12 (0.1%)	12 (0.1%)	12 (0.0%)	13 (0.1%)	0.12
Asthma, n (%)	3486 (4.1%)	561 (3.7%)	838 (4.5%)	1265 (3.6%)	822 (4.9%)	<.001
Bronchiectasis, n (%)	88 (0.1%)	16 (0.1%)	18 (0.1%)	36 (0.1%)	18 (0.1%)	0.99
Cannabis disorder, n (%)	2235 (2.6%)	393 (2.6%)	576 (3.1%)	869 (2.5%)	397 (2.4%)	<.001
Chronic back pain, n (%)	7348 (8.6%)	1049 (6.8%)	2055 (11.1%)	2587 (7.4%)	1657 (9.9%)	<.001
Chronic obstructive pulmonary disease, n (%)	3714 (4.3%)	428 (2.8%)	1078 (5.8%)	1461 (4.2%)	747 (4.5%)	<.001
Epilepsy, n (%)	1625 (1.9%)	257 (1.7%)	376 (2.0%)	653 (1.9%)	338 (2.0%)	0.066
Fibromyalgia/Myalgia, n (%)	5971 (7.0%)	918 (6.0%)	1586 (8.6%)	2196 (6.3%)	1271 (7.6%)	<.001
Gastro-oesophageal reflux, n (%)	5352 (6.3%)	647 (4.2%)	1419 (7.7%)	2084 (6.0%)	1202 (7.2%)	<.001
Hallucinogen use disorder, n (%)	59 (0.1%)	8 (0.1%)	15 (0.1%)	12 (0.0%)	24 (0.1%)	0.0001
Herpes zoster, n (%)	143 (0.2%)	21 (0.1%)	38 (0.2%)	56 (0.2%)	28 (0.2%)	0.47
Human immunodeficiency virus, n (%)	461 (0.5%)	92 (0.6%)	88 (0.5%)	188 (0.5%)	93 (0.6%)	0.47
Hyperlipidaemia, n (%)	8697 (10.2%)	1032 (6.7%)	2189 (11.8%)	3377 (9.7%)	2097 (12.5%)	<.001
Metabolic diseases, n (%)	5314 (6.2%)	754 (4.9%)	1319 (7.1%)	2196 (6.3%)	1045 (6.2%)	<.001
Osteoarthritis, n (%)	3465 (4.1%)	388 (2.5%)	956 (5.2%)	1337 (3.8%)	784 (4.7%)	<.001
Osteoporosis, n (%)	778 (0.9%)	107 (0.7%)	198 (1.1%)	245 (0.7%)	228 (1.4%)	<.001
Other chronic musculoskeletal problems, n (%)	12067 (14.1%)	1833 (12.0%)	3261 (17.6%)	4224 (12.1%)	2746 (16.4%)	<.001
Psoriasis, n (%)	258 (0.3%)	36 (0.2%)	62 (0.3%)	85 (0.2%)	75 (0.4%)	0.0003
Renal disease, n (%)	2858 (3.3%)	441 (2.9%)	585 (3.2%)	1237 (3.5%)	595 (3.6%)	0.0003
Rheumatoid arthritis, n (%)	1427 (1.7%)	201 (1.3%)	340 (1.8%)	553 (1.6%)	333 (2.0%)	<.001
Schizophrenia, n (%)	1253 (1.5%)	256 (1.7%)	309 (1.7%)	465 (1.3%)	223 (1.3%)	0.0011
Thyroid, n (%)	3092 (3.6%)	447 (2.9%)	800 (4.3%)	1104 (3.2%)	741 (4.4%)	<.001
Tobacco disorder, n (%)	12666 (14.8%)	1783 (11.6%)	3499 (18.9%)	5085 (14.6%)	2297 (13.7%)	<.001
Tuberculosis, n (%)	32 (0.0%)	5 (0.0%)	4 (0.0%)	17 (0.0%)	6 (0.0%)	0.47

Forty-four samples with missing state information were excluded. Data are No. (%) or mean (SD). Avg UDT=the mean of quantitative urine drug testing values.

[a]Kruskal-Wallis tests were performed to compare all region subcohorts.

[b]Conditions were assessed within three months prior the index date.

Statistical Analysis

To evaluate the causal association between illicit-source fentanyl initiation and 30-day opioid-related harmful outcomes, we estimated hazard ratios (HRs) using Cox proportional hazards models. The exposure was illicit-source fentanyl initiation at the index date (the initial fentanyl exposure date). Individuals with a positive UDT at the index date were classified as having illicit-source fentanyl initiation (exposure), whereas those with a fentanyl medication record at the index date were classified as having medical-source fentanyl initiation (no exposure). The outcome was the occurrence of an opioid-related harmful outcome (e.g., opioid dependence) within 30 days after the index date. Individuals who experienced the outcome were coded as events at the observed time-to-event, whereas those without the outcome were coded as censored at day 30. Individuals with any prior opioid-related harmful outcomes (overdose, abuse, or dependence) were excluded for the association analysis to ensure assessment of incident outcomes.

Subgroup analysis

Supplementary Table 8. Adjusted associations of illicit-source versus medical-source fentanyl initiation with opioid-related harmful outcomes in the illicit-source fentanyl exposure cohort and subgroups stratified by substance use disorders, mental health conditions, and chronic conditions.

Adjusted hazard ratios (HRs) with 95% confidence intervals and *P* values are presented for nonfatal opioid overdose, opioid abuse, and opioid dependence, with medical-source fentanyl initiation as the reference group. Positive subgroups include patients with prior corresponding conditions, whereas negative subgroups include those without. Patients with any prior opioid-related harmful outcomes were excluded. No unbalanced covariates remained after stabilized inverse probability weighting in any analysis.

Subgroup		# Patients	Overdose	Abuse	Dependence
**All**		121515	2.84 (2.46–3.28); <.001	1.53 (1.40–1.68); <.001	2.25 (2.09–2.42); <.001
**Substance**	Positive	53616	2.69 (2.26–3.20); <.001	1.92 (1.71–2.16); <.001	2.75 (2.50–3.03); <.001
	Negative	67899	3.08 (2.41–3.93); <.001	1.25 (1.10–1.43); .0008	1.97 (1.77–2.19); <.001
**Mental**	Positive	43943	2.80 (2.25–3.49); <.001	2.42 (2.06–2.83); <.001	2.81 (2.49–3.16); <.001
	Negative	77572	2.79 (2.31–3.37); <.001	1.21 (1.08–1.35); 0.0006	2.01 (1.83–2.20); <.001
**Chronic**	Positive	72516	3.31 (2.80–3.92); <.001	2.15 (1.92–2.40); <.001	2.86 (2.61–3.13); <.001
	Negative	48999	2.44 (1.89–3.15); <.001	1.05 (0.92–1.21); .461	1.82 (1.62–2.05); <.001

Sensitivity Analysis

Supplementary Table 9. Sensitivity analysis using alternative UDT screening windows (48, 72, and 96 hours) for exposure classification in the entire cohort, comparing illicit- versus medical-source fentanyl initiation (reference). Adjusted hazard ratios (HRs) with 95% confidence intervals and *P* values are presented for nonfatal opioid overdose, opioid abuse, and opioid dependence. Patients with any prior opioid-related harmful outcomes were excluded. No unbalanced covariates remained after stabilized inverse probability weighting in any analysis.

	# Patients		Entire cohort		
**Hours**	**Medical**	**Illicit**	**Overdose**	**Abuse**	**Dependence**
48	84753	210975	2.99 (2.71–3.29); <.001	1.96 (1.84–2.10); <.001	3.05 (2.89–3.22); <.001
72	85535	210193	2.99 (2.71–3.29); <.001	1.96 (1.84–2.10); <.001	3.05 (2.89–3.22); <.001
96	86037	209691	2.99 (2.71–3.29); <.001	1.96 (1.84–2.10); <.001	3.05 (2.89–3.22); <.001

Supplementary Table 10. Sensitivity analysis using alternative follow-up windows (30, 60, 90, and 180 days) for outcome classification in the entire and the illicit-source fentanyl exposure cohorts, comparing illicit- versus medical-source fentanyl initiation (reference). Adjusted hazard ratios (HRs) with 95% confidence intervals and *P* values are presented for opioid overdose, abuse, and dependence. Patients with any prior opioid-related harmful outcomes were excluded. No unbalanced covariates remained after stabilized inverse probability weighting in any analysis.

	**Entire cohort**		
**Window**	**Overdose**	**Abuse**	**Dependence**
30	2.99 (2.71–3.29); <.001	1.96 (1.84–2.10); <.001	3.05 (2.89–3.22); <.001
60	2.61 (2.40–2.84); <.001	1.94 (1.83–2.06); <.001	2.77 (2.64–2.91); <.001
90	2.38 (2.21–2.57); <.001	1.90 (1.80–2.01); <.001	2.57 (2.46–2.69); <.001
180	1.99 (1.86–2.12); <.001	1.71 (1.63–1.80); <.001	2.17 (2.09–2.26); <.001
	**Illicit-source cohort**		
30	2.84 (2.46–3.28); <.001	1.53 (1.40–1.68); <.001	2.25 (2.09–2.42); <.001
60	1.92 (1.72–2.15); <.001	1.39 (1.29–1.50); <.001	1.94 (1.82–2.07); <.001
90	1.62 (1.47–1.78); <.001	1.28 (1.20–1.37); <.001	1.74 (1.64–1.84); <.001
180	1.20 (1.12–1.30); <.001	1.06 (1.00–1.12); .058	1.37 (1.31–1.44); <.001

Evaluation of baseline covariate balance

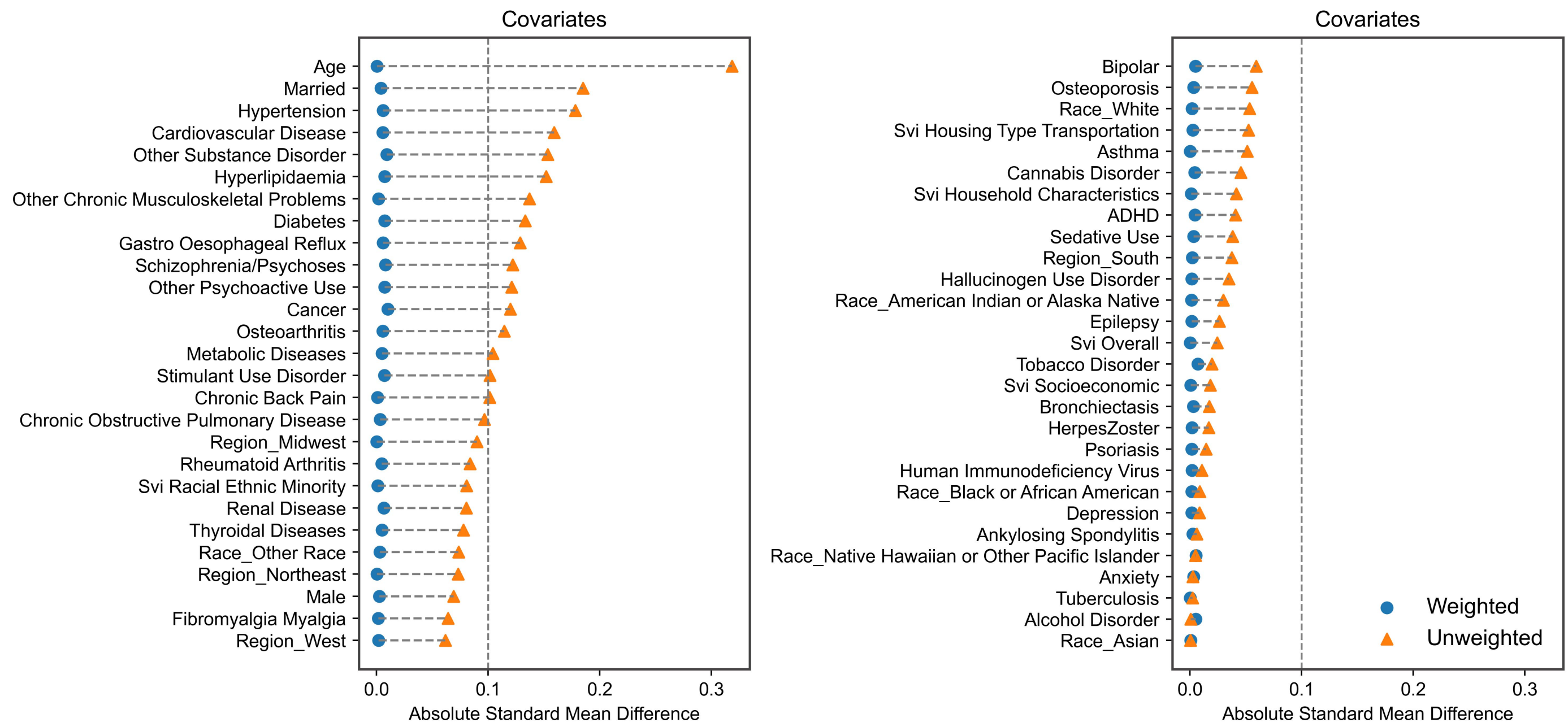

A

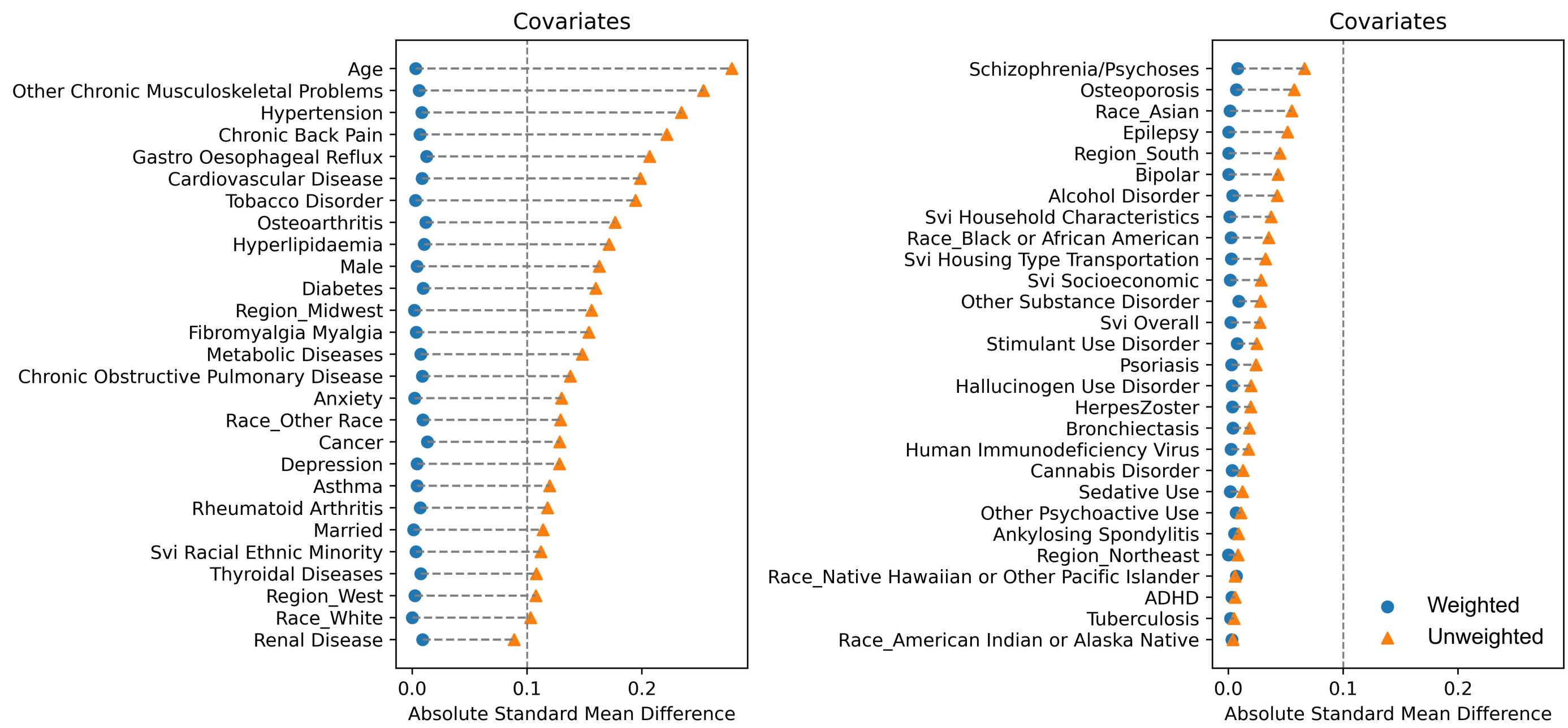

B

Supplementary Figure 2. Absolute standardized mean differences (SMD) before and after weighting for A. the entire cohort and B. the illicit-source fentanyl exposure cohort. Values of |SMD| ≤ 0.10 indicate adequate balance.[6] Analyses were considered balanced if the proportion of unbalanced covariates was ≤ 2% of all covariates. All covariates met the balance criteria.

Supplementary Table 11. Absolute standardized mean differences (SMD) before and after weighting for the entire cohort. Values of |SMD| ≤ 0.10 indicate adequate balance.[6] Analyses were considered balanced if the proportion of unbalanced covariates was ≤ 2% of all covariates. All covariates met the balance criteria.

Covariate	Weighted	Unweighted	Covariate	Weighted	Unweighted
ADHD	0.00454	0.04076	Other Chronic Musculoskeletal Problems	0.00171	0.13693
Age	0.00035	0.31850	Other Psychoactive Use	0.00734	0.12094
Alcohol Disorder	0.00515	0.00063	Other Substance Disorder	0.00929	0.15336
Ankylosing Spondylitis	0.00260	0.00620	Psoriasis	0.00160	0.01435
Anxiety	0.00333	0.00264	Race - American Indian or Alaska Native	0.00151	0.02986
Asthma	0.00027	0.05125	Race - Asian	0.00062	0.00050
Bipolar	0.00504	0.05929	Race - Black or African American	0.00156	0.00872
Bronchiectasis	0.00294	0.01724	Race - Native Hawaiian or Other Pacific Islander	0.00531	0.00500
Cancer	0.01002	0.11987	Race - Other Race	0.00299	0.07354
Cannabis Disorder	0.00416	0.04561	Race - White	0.00183	0.05336
Cardiovascular Disease	0.00573	0.15903	Region - Midwest	0.00026	0.08984
Chronic Back Pain	0.00083	0.10130	Region - Northeast	0.00036	0.07310
Chronic Obstructive Pulmonary Disease	0.00318	0.09669	Region - South	0.00201	0.03742
Depression	0.00157	0.00849	Region - West	0.00187	0.06165
Diabetes	0.00733	0.13312	Renal Disease	0.00647	0.08038
Epilepsy	0.00172	0.02636	Rheumatoid Arthritis	0.00464	0.08372
Fibromyalgia Myalgia	0.00158	0.06408	Schizophrenia/Psychoses	0.00791	0.12189
Gastro Oesophageal Reflux	0.00595	0.12867	Sedative Use	0.00325	0.03822
Hallucinogen Use Disorder	0.00172	0.03491	Stimulant Use Disorder	0.00712	0.10155
Herpes Zoster	0.00188	0.01680	SVI - Household Characteristics	0.00108	0.04144
Human Immunodeficiency Virus	0.00190	0.01059	SVI - Housing Type Transportation	0.00262	0.05249
Hyperlipidaemia	0.00729	0.15199	SVI - Overall	0.00019	0.02442
Hypertension	0.00585	0.17810	SVI - Racial Ethnic Minority	0.00121	0.08079
Male	0.00256	0.06907	SVI - Socioeconomic	0.00073	0.01828
Married	0.00391	0.18491	Thyroidal Diseases	0.00483	0.07781
Metabolic Diseases	0.00481	0.10420	Tobacco Disorder	0.00716	0.01961
Osteoarthritis	0.00553	0.11443	Tuberculosis	0.00031	0.00229
Osteoporosis	0.00320	0.05564			

Supplementary Table 12. Absolute standardized mean differences (SMD) before and after weighting for the illicit-source fentanyl exposure cohort. Values of |SMD| ≤ 0.10 indicate adequate balance.[6] Analyses were considered balanced if the proportion of unbalanced covariates was ≤ 2% of all covariates. All covariates met the balance criteria.

Covariate	Weighted	Unweighted	Covariate	Weighted	Unweighted
ADHD	0.00304	0.00576	Other Chronic Musculoskeletal Problems	0.00601	0.25355
Age	0.00299	0.27820	Other Psychoactive Use	0.00670	0.01096
Alcohol Disorder	0.00381	0.04246	Other Substance Disorder	0.00918	0.02793
Ankylosing Spondylitis	0.00526	0.00873	Psoriasis	0.00292	0.02417
Anxiety	0.00216	0.12992	Race - American Indian or Alaska Native	0.00295	0.00395
Asthma	0.00433	0.11974	Race - Asian	0.00145	0.05513
Bipolar	0.00026	0.04328	Race - Black or African American	0.00229	0.03506
Bronchiectasis	0.00402	0.01821	Race - Native Hawaiian or Other Pacific Islander	0.00690	0.00592
Cancer	0.01330	0.12838	Race - Other Race	0.00934	0.12902
Cannabis Disorder	0.00334	0.01286	Race - White	0.00013	0.10300
Cardiovascular Disease	0.00866	0.19858	Region - Midwest	0.00198	0.15606
Chronic Back Pain	0.00674	0.22169	Region - Northeast	0.00003	0.00839
Chronic Obstructive Pulmonary Disease	0.00885	0.13754	Region - South	0.00021	0.04488
Depression	0.00417	0.12826	Region - West	0.00244	0.10762
Diabetes	0.00945	0.15973	Renal Disease	0.00912	0.08868
Epilepsy	0.00018	0.05159	Rheumatoid Arthritis	0.00698	0.11778
Fibromyalgia Myalgia	0.00345	0.15386	Schizophrenia/Psychoses	0.00804	0.06630
Gastro Oesophageal Reflux	0.01254	0.20668	Sedative Use	0.00165	0.01225
Hallucinogen Use Disorder	0.00320	0.01975	Stimulant Use Disorder	0.00768	0.02467
Herpes Zoster	0.00364	0.01948	SVI - Household Characteristics	0.00133	0.03720
Human Immunodeficiency Virus	0.00243	0.01762	SVI - Housing Type Transportation	0.00265	0.03227
Hyperlipidaemia	0.01056	0.17125	SVI - Overall	0.00216	0.02752
Hypertension	0.00835	0.23434	SVI - Racial Ethnic Minority	0.00334	0.11204
Male	0.00427	0.16285	SVI - Socioeconomic	0.00172	0.02851
Married	0.00125	0.11390	Thyroidal Diseases	0.00748	0.10808
Metabolic Diseases	0.00747	0.14811	Tobacco Disorder	0.00279	0.19438
Osteoarthritis	0.01179	0.17676	Tuberculosis	0.00190	0.00501
Osteoporosis	0.00693	0.05730			

Model Specifications

Supplementary Table 13. Estimated coefficients from the logistic regression model used for propensity score estimation across all covariates in the entire cohort.

Covariate	Coefficient	Covariate	Coefficient
ADHD	0.00915	Other Chronic Musculoskeletal Problems	-0.15363
Age	-0.01907	Other Psychoactive Use	0.07497
Alcohol Disorder	-0.12442	Other Substance Disorder	0.53807
Ankylosing Spondylitis	0.02657	Psoriasis	0.08609
Anxiety	0.07758	Race - American Indian or Alaska Native	0.08858
Asthma	-0.19832	Race - Asian	-0.10756
Bipolar	0.12314	Race - Black or African American	-0.06175
Bronchiectasis	0.02912	Race - Native Hawaiian or Other Pacific Islander	-0.18377
Cancer	-0.42065	Race - Other Race	0.19134
Cannabis Disorder	-0.16143	Race - White	0.00701
Cardiovascular Disease	-0.12954	Region - Midwest	-0.24154
Chronic Back Pain	-0.03406	Region - Northeast	0.19905
Chronic Obstructive Pulmonary Disease	0.02488	Region - South	-0.09159
Depression	0.08441	Region - West	0.06793
Diabetes	-0.16231	Renal Disease	0.10372
Epilepsy	-0.17083	Rheumatoid Arthritis	-0.09444
Fibromyalgia Myalgia	0.01807	Schizophrenia/Psychoses	0.57864
Gastro Oesophageal Reflux	-0.19284	Sedative Use	0.22316
Hallucinogen Use Disorder	0.04108	Stimulant Use Disorder	0.01776
Herpes Zoster	0.14379	SVI - Household Characteristics	-0.35605
Human Immunodeficiency Virus	-0.07206	SVI - Housing Type Transportation	0.11572
Hyperlipidaemia	0.09141	SVI - Overall	0.06245
Hypertension	-0.02977	SVI - Racial Ethnic Minority	0.37852
Male	0.15840	SVI - Socioeconomic	-0.07515
Married	-0.28997	Thyroidal Diseases	0.04328
Metabolic Diseases	-0.16357	Tobacco Disorder	-0.07334
Osteoarthritis	0.09363	Tuberculosis	-0.01508
Osteoporosis	0.23527		

Supplementary Table 14. Estimated coefficients from the logistic regression model used for propensity score estimation across all covariates in the illicit-source fentanyl exposure cohort.

Covariate	Coefficient	Covariate	Coefficient
ADHD	0.09638	Other Chronic Musculoskeletal Problems	-0.26915
Age	-0.01356	Other Psychoactive Use	-0.04830
Alcohol Disorder	-0.00517	Other Substance Disorder	0.27176
Ankylosing Spondylitis	0.05778	Psoriasis	0.16014
Anxiety	0.02463	Race - American Indian or Alaska Native	-0.23106
Asthma	-0.19360	Race - Asian	0.42547
Bipolar	0.00835	Race - Black or African American	-0.07556
Bronchiectasis	0.05034	Race - Native Hawaiian or Other Pacific Islander	-0.27846
Cancer	-0.46669	Race - Other Race	0.37257
Cannabis Disorder	-0.07729	Race - White	-0.20550
Cardiovascular Disease	-0.13490	Region - Midwest	-0.33069
Chronic Back Pain	-0.15538	Region - Northeast	0.02972
Chronic Obstructive Pulmonary Disease	0.08191	Region - South	0.08975
Depression	0.00896	Region - West	0.21868
Diabetes	-0.23159	Renal Disease	0.05818
Epilepsy	-0.12691	Rheumatoid Arthritis	-0.18876
Fibromyalgia Myalgia	0.02535	Schizophrenia/Psychoses	0.51583
Gastro Oesophageal Reflux	-0.30302	Sedative Use	0.29676
Hallucinogen Use Disorder	0.09056	Stimulant Use Disorder	0.08710
Herpes Zoster	0.27755	SVI - Household Characteristics	-0.28149
Human Immunodeficiency Virus	-0.30868	SVI - Housing Type Transportation	-0.00975
Hyperlipidaemia	0.20549	SVI - Overall	-0.06538
Hypertension	-0.15879	SVI - Racial Ethnic Minority	0.30663
Male	0.40554	SVI - Socioeconomic	0.02313
Married	-0.13249	Thyroidal Diseases	0.05136
Metabolic Diseases	-0.08915	Tobacco Disorder	-0.43660
Osteoarthritis	-0.05239	Tuberculosis	-0.00721
Osteoporosis	0.35411		

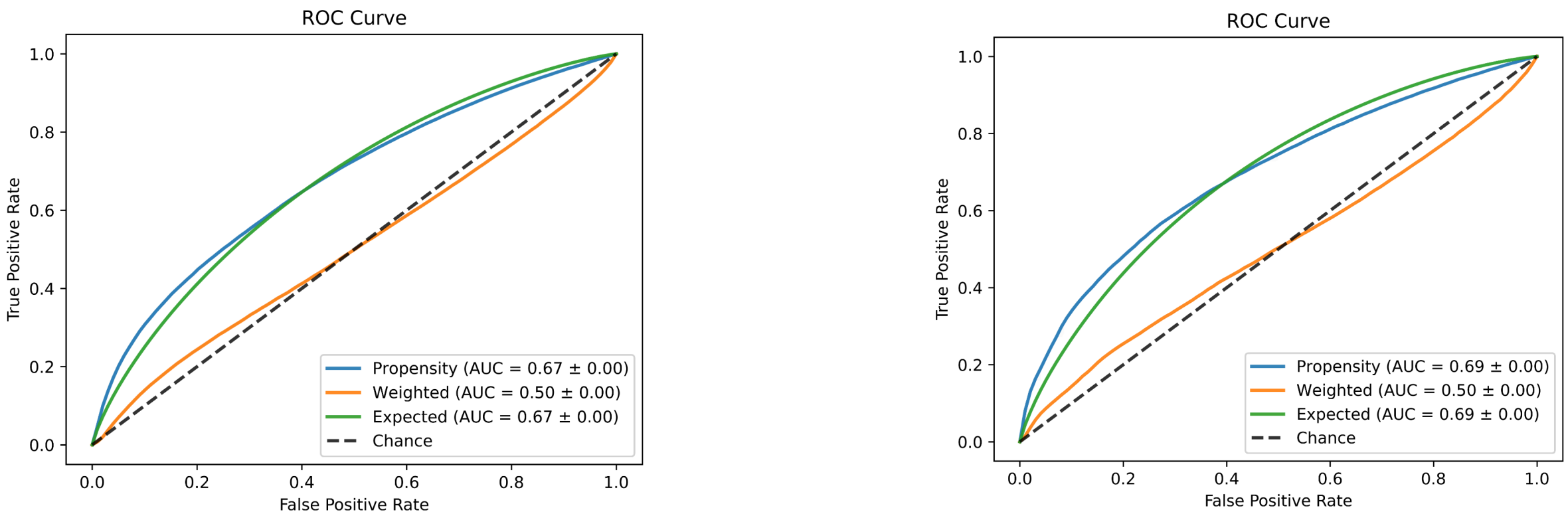

A B

Supplementary Figure 3. The ROC curves of the unweighted propensity (blue), the expected propensity (green), and the inverse-probability weighted propensity (orange) for A. the entire cohort and B. the illicit-source fentanyl exposure cohort.

Reference

1. Centers for Disease Control and Prevention. Geographic division or region. July 30, 2024. Accessed December 12, 2025. https://www.cdc.gov/nchs/hus/sources-definitions/geographic-region.htm
2. Wilson JD, Abebe KZ, Kraemer K, et al. Trajectories of opioid use following first opioid prescription in opioid-naive youths and young adults. *JAMA Netw Open*. 2021;4(4):e214552-e214552.
3. Zhang P, Tossone K, Ashmead R, et al. Examining differences in retention on medication for opioid use disorder: An analysis of Ohio Medicaid data. *J Subst Abuse Treat*. 2022;136:108686.
4. Binswanger IA, Shetterly SM, Xu S, et al. Opioid dose trajectories and associations with mortality, opioid use disorder, continued opioid therapy, and health plan disenrollment. *JAMA Netw Open*. 2022;5(10):e2234671-e2234671.
5. Linnet K, Gudmundsson LS, Birgisdottir FG, et al. Multimorbidity and use of hypnotic and anxiolytic drugs: cross-sectional and follow-up study in primary healthcare in Iceland. *BMC Fam Pract*. 2016;17:69. doi:10.1186/s12875-016-0469-0
6. Austin PC. Using the Standardized Difference to Compare the Prevalence of a Binary Variable Between Two Groups in Observational Research. *Commun Stat - Simul Comput*. 2009;38(6):1228-1234. doi:10.1080/03610910902859574
7. Owens PL, Weiss AJ, Barrett ML. Hospital Burden of Opioid-Related Inpatient Stays: Metropolitan and Rural Hospitals, 2016. *Healthcare Cost and Utilization Project (HCUP) Statistical Briefs*. 2020. PMID: 32658442.
8. El Ibrahimi S, Hendricks MA, Little K, Ritter GA, Flores D, Loy B, Wright D, Weiner SG. The association between community social vulnerability and prescription opioid availability with individual opioid overdose. *Drug and alcohol dependence*. 2023 Nov 1;252:110991.
